# Supplementary material for: Efficient all-small-molecule organic solar cells processed with non-halogen solvent
Source: Nat Commun. 2024 Mar 2;15:1946. doi: 10.1038/s41467-024-46144-8 (PMC10908865; doi:10.1038/s41467-024-46144-8)
Supplement: Supplementary file 1 — Supplementary Information [file 41467_2024_46144_MOESM1_ESM.pdf]

Supplementary Information

Efficient all-small-molecule organic solar cells processed with non-halogen solvent

Wei Gao[1,#], Ruijie Ma[2,#,], Top Archie Dela Peña[3,4,#], Cenqi Yan[5], Hongxiang Li[5,*], Mingjie Li[3], Jiaying Wu[4], Pei Cheng[5], Cheng Zhong[6], Zhanhua Wei[1], Alex K.-Y. Jen[7,8,*], Gang Li[2,*]*

[1]Xiamen Key Laboratory of Optoelectronic Materials and Advanced Manufacturing, Institute of Luminescent Materials and Information Displays, College of Materials Science and Engineering, Huaqiao University, Xiamen 361021, China

[2]Department of Electrical and Electronic Engineering, Research Institute for Smart Energy (RISE), The Hong Kong Polytechnic University, Hung Hom, Kowloon, Hong Kong 999077, China

[3]Department of Applied Physics, The Hong Kong Polytechnic University, Hong Kong 999077, China

[4]Advanced Materials Thrust, Function Hub, The Hong Kong University of Science and Technology, Nansha, Guangzhou 511442, China

[5]College of Polymer Science and Engineering, State Key Laboratory of Polymer Materials Engineering, Sichuan University, Chengdu 610064, China

[6]Department of Chemistry, Hubei Key Lab on Organic and Polymeric Optoelectronic Materials, Wuhan University, Wuhan, 430072, China

[7]Department of Materials Science and Engineering, City University of Hong Kong, Kowloon, 999077, Hong Kong, China

[8]Hong Kong Institute for Clean Energy, City University of Hong Kong, Kowloon 999077, Hong Kong, China

[#]These authors contributed equally

*Corresponding authors: ruijie.ma@polyu.edu.hk; lihongxiang@scu.edu.cn; alexjen@cityu.edu.hk; gang.w.li@polyu.edu.hk

Supplementary Methods

Materials synthesis

All solvents and reagents were used as received from commercial sources and used without further purification. Compound **1**, Compound **2**, 3-butyl-2-thioxothiazolidin-4-one, 3-(2-ethylhexyl)-2-thioxothiazolidin-4-one, small-molecule donor (SMD) BM-Cl and small-molecule acceptor (SMA) BO-4Cl were synthesized according to reported methods.[1-3] ^{1}H NMR and ^{13}C NMR spectra were recorded on Bruker AVANCE (400 MHz). The matrix-assisted laser desorption/ionization time of flight mass spectrometry (MALDI-TOF-MS) were

performed on Tempo LC MALDI spotting System (ABI).

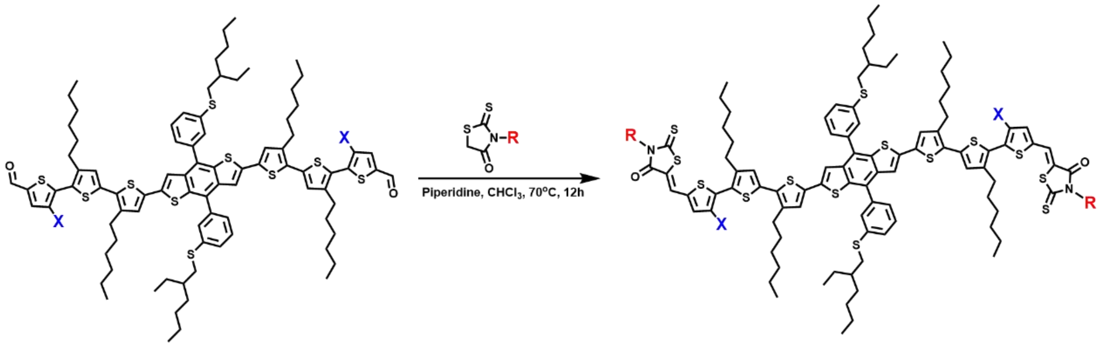

Supplementary Figure 1 Synthetic routes for SMDs of BM-Cl, BM-ClEH and BM-HEH.

Synthesis of (5Z,5'Z)-5,5'-(((4,8-bis(3-((2-ethylhexyl)thio)phenyl)benzo[1,2-b:4,5-b']dithiophene-2,6-diyl)bis(3-chloro-3',3''-dihexyl-[2,2':5',2''-terthiophene]-5'',5-diyl))bis(methaneylylidene))bis(3-(2-ethylhexyl)-2-thioxothiazolidin-4-one) (**BM-ClEH**)[1]: To a 100 mL round bottom flask, compound **1** (300 mg, 0.189 mmol) and 3-(2-ethylhexyl)-2-thioxothiazolidin-4-one (280 mg, 1.13 mmol) were added under N_2 protection. Then, deoxidized chloroform (30 ml) was added and stirred for a while when piperidine (3 drop) was added. The mixture was kept stirring at 70°C for 12 h. After cooling to room temperature, 50 ml methanol was added and the precipitate was collected by filtration. The residue was purified by column chromatography on silica gel using a mixture solvent as eluent (hexane/chloroform, v/v = 1/1) to give a dark solid (293 mg, 76%). ^{1}H NMR (400 MHz, $CDCl_3$), δ (ppm): 7.72 (s, 2H), 7.66 (s, 2H), 7.48-7.52 (m, 6H), 7.32 (s, 2H), 7.30 (s, 2H), 7.07 (s, 2H), 7.03 (s, 2H), 4.04 (d, 4H), 3.02 (d, 4H), 2.75 (t, 4H), 2.67 (t, 4H), 2.04-2.07 (m, 2H), 1.62-1.71 (m, 10H), 1.29-1.34 (m, 56H), 0.83-0.94 (m, 36H). MALDI-TOF-MS m/z: [M] calcd. for $C_{110}H_{138}Cl_2N_2O_2S_{14}$, 2037.62588, found 2037.87573.

Synthesis of (5Z,5'Z)-5,5'-(((4,8-bis(3-((2-ethylhexyl)thio)phenyl)benzo[1,2-b:4,5-b']dithiophene-2,6-diyl)bis(3',3''-dihexyl-[2,2':5',2''-terthiophene]-5'',5-diyl))bis(methaneylylidene))bis

(3-(2-ethylhexyl)-2-thioxothiazolidin-4-one) (**BM-HEH**)[1]: To a 100 mL round bottom flask, compound **2** (300 mg, 0.198 mmol) and 3-(2-ethylhexyl)-2-thioxothiazolidin-4-one (290 mg, 1.19 mmol) were added under N_2 protection. Then, deoxidized chloroform (30 ml) was added and stirred for a while when piperidine (3 drop) was added. The mixture was kept stirring at 70°C for 12 h. After cooling to room temperature, 50 ml methanol was added and the precipitate was collected by filtration. The residue was purified by column chromatography on silica gel using a mixture solvent as eluent (hexane/chloroform, v/v = 1/1) to give a red solid (312 mg, 80%). ^1H NMR (400 MHz, $CDCl_3$), δ (ppm): 7.83 (s, 2H), 7.65 (s, 2H), 7.48-7.53 (m, 6H), 7.36 (d, 2H), 7.30 (s, 2H), 7.20 (d, 2H), 7.05 (s, 2H), 6.98 (s, 2H), 4.03 (d, 4H), 3.03 (d, 4H), 2.80 (t, 4H), 2.74 (t, 4H), 2.04-2.09 (m, 2H), 1.62-1.73 (m, 10H), 1.29-1.36 (m, 56H), 0.83-0.95 (m, 36H). MALDI-TOF-MS m/z: [M] calcd. for $C_{110}H_{140}N_2O_2S_{14}$, 1968.70047, found 1968.69983.

Solubility test

Solubilities of three SMDs were estimated by carefully adding quantitative amounts of THF solvent to dissolve a certain amount of compound: 5 mg compound was placed in a 1.5 ml glass bottle with good airtightness. After adding 50 μL THF with a pipette gun, the bottle was heated with a metal heating module at 60°C for 2 hours, then cool and stand still for 5 hours to observe whether there is any compound precipitation. This operation was repeated, and it was found that after adding THF for the third time, BM-ClEH and BM-HEH could be completely dissolved and no compound was precipitated after standing overnight. And BM-Cl still has a lot of undissolved. Aa a result, the final solubility of BM-ClEH and BM-HEH is estimated to be between 30 and 50 mg.

DFT calculations

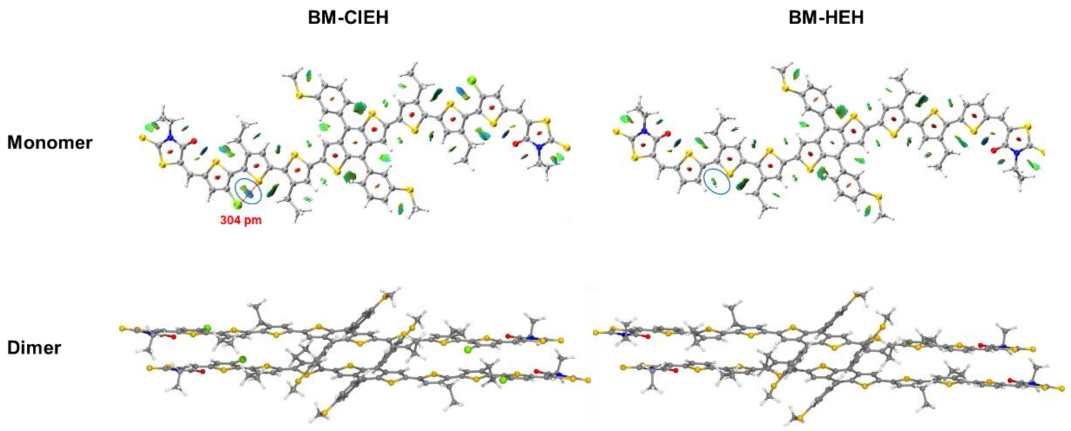

Supplementary Figure 2 DFT calculation results for BM-ClEH and BM-HEH monomer and dimer.

DSC measurements

Crystalline properties of three SMDs were characterized by differential scanning calorimetry (DSC) measurements on METTLER TOLEDO TGA/DGA3+. The mass of sample is ~3.0 mg, and heating rate and cooling rate are both 5°C/min.

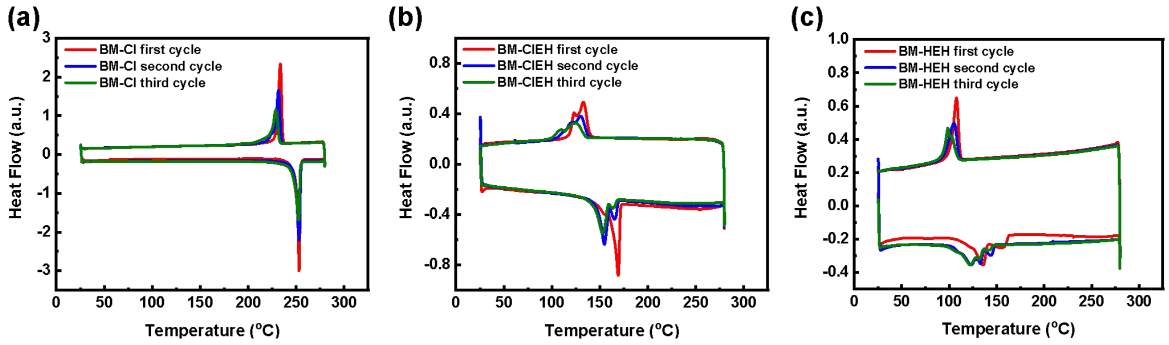

Supplementary Figure 3 Multi-turn DSC scan curves for (a) BM-Cl; (b) BM-ClEH and (c) BM-HEH.

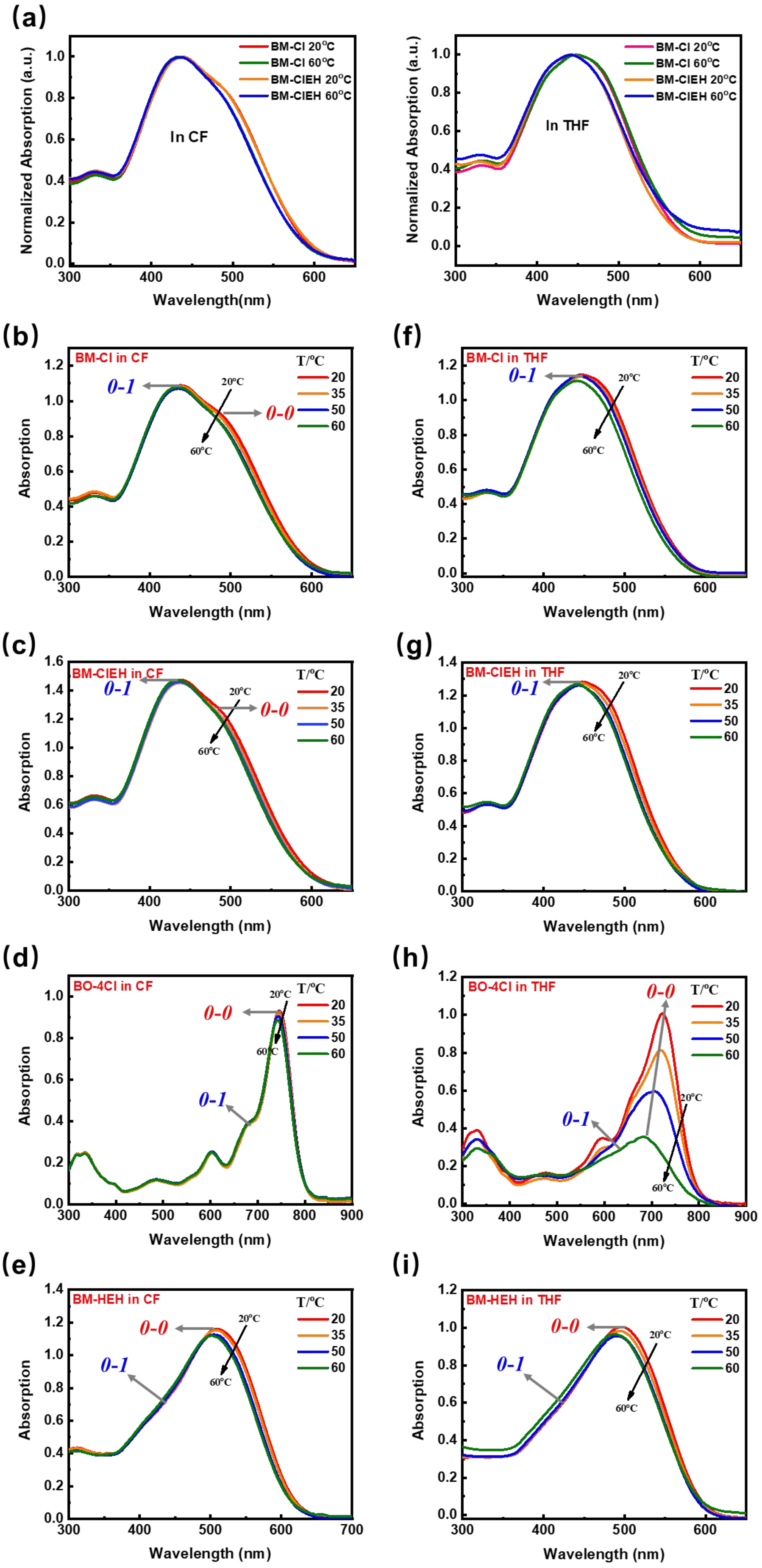
(a)
In CF
BM-Cl 20°C
BM-Cl 60°C
BM-ClEH 20°C
BM-ClEH 60°C
Normalized Absorption (a.u.)
Wavelength(nm)
In THF
Wavelength (nm)
(b)
BM-Cl in CF
(c)
BM-ClEH in CF
(d)
BO-4Cl in CF
(e)
BM-HEH in CF
(f)
BM-Cl in THF
(g)
BM-ClEH in THF
(h)
BO-4Cl in THF
(i)
BM-HEH in THF
T/°C
20
35
50
60
20°C
60°C
0-0
0-1
Absorption
Wavelength (nm)

Supplementary Figure 4 (a) Absorption spectra comparison between BM-Cl and BM-ClEH in chloroform (CF) and tetrahydrofuran (THF) at 20 and 60 °C. Temperature-variable UV-vis absorption spectra of solutions for (b) BM-Cl in CF; (c) BM-ClEH in CF; (d) BO-4Cl in CF; (e) BM-HEH in CF; (f) BM-Cl in THF; (g) BM-ClEH in THF; (h) BO-4Cl in THF and (i) BM-HEH in THF.

Temperature-variable NMR test

The concentration of test sample is 5 mg/mL in $CDCl_3$. Range of test temperature for NMR (Bruker AVANCE 400 MHz) spectra is from 20 °C to 56 °C, lower than boiling point of deuterated reagent. And the ^1H NMR signals were acquired every 4 °C.

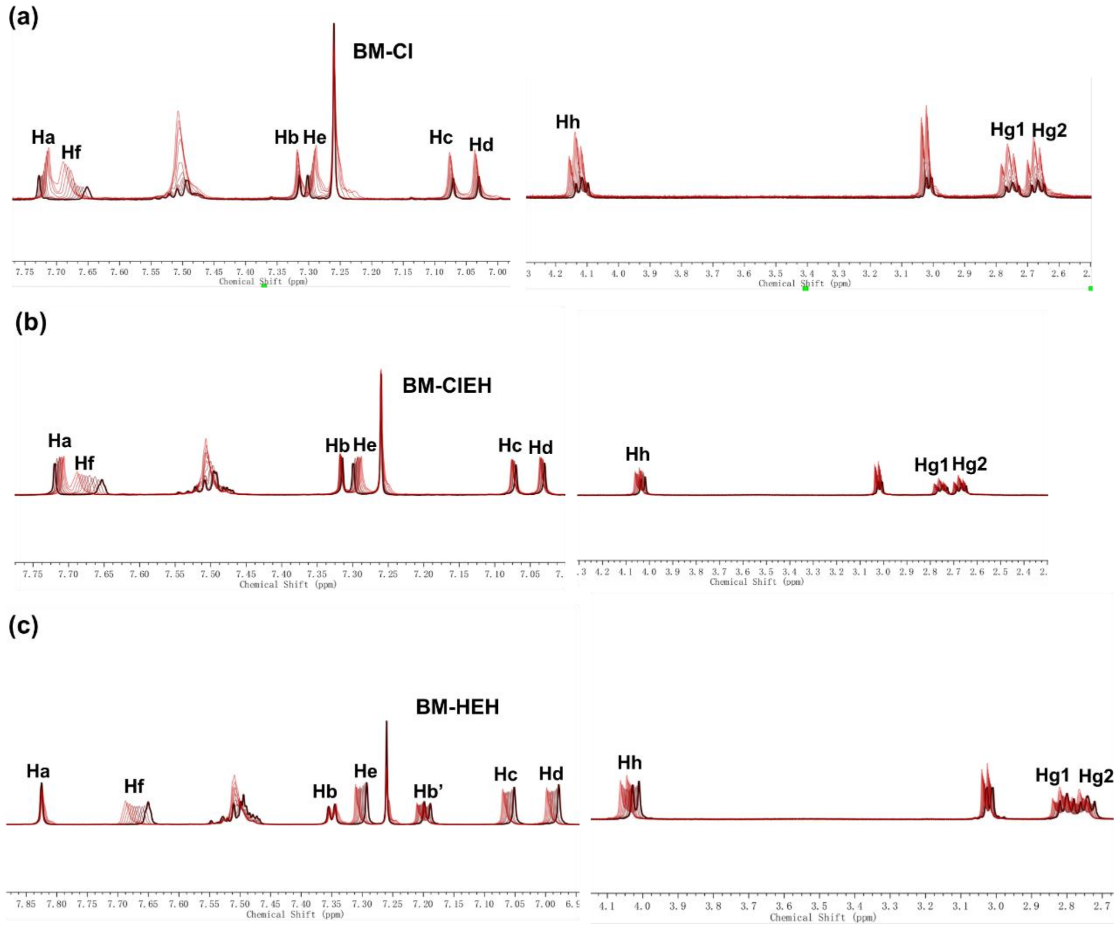

Supplementary Figure 5 Temperature-variable ^1H NMR spectra of (a) BM-Cl; (b) BM-ClEH and (c) BM-HEH.

Supplementary Table 1 Chemical shifts of different H atoms of BM-Cl under different temperature.

°C/ppm	Ha	Hb	Hc	Hd	He	Hh	Hf	Hg1	Hg2
20	7.7308	7.3175	7.0735	7.0329	7.3047	4.1191	7.6546	2.75	2.669
24	7.7283	7.318	7.0746	7.0339	7.3026	4.1225	7.6607	2.7534	2.6715
28	7.7266	7.3184	7.0753	7.0346	7.3012	4.1252	7.6652	2.7548	2.6729
32	7.7248	7.3188	7.0759	7.0357	7.2996	4.1277	7.6689	2.7565	2.6748
36	7.7233	7.3192	7.0766	7.036	7.2983	4.1303	7.6731	2.7589	2.6762
40	7.7214	7.3194	7.077	7.0365	7.2967	4.1326	7.6773	2.7601	2.6779
44	7.7192	7.3197	7.0773	7.037	7.2947	4.1339	7.6804	2.7611	2.6783
48	7.7178	7.3202	7.0781	7.0377	7.2935	4.1366	7.6847	2.7635	2.68
52	7.7165	7.3207	7.0787	7.0385	7.2924	4.139	7.6886	2.7648	2.682
56	7.7151	7.3213	7.0794	7.0395	7.2911	4.1415	7.6922	2.7664	2.6837

Supplementary Table 2 Chemical shifts of different H atoms of BM-ClEH under different temperature .

°C/ppm	Ha	Hb	Hc	Hd	He	Hh	Hf	Hg1	Hg2
20	7.723	7.3173	7.0732	7.0328	7.3028	4.0386	7.656	2.7502	2.6691
24	7.721	7.318	7.0743	7.0339	7.301	4.0427	7.6616	2.7531	2.6713
28	7.7189	7.3183	7.0749	7.0345	7.2991	4.0453	7.6655	2.7547	2.6728
32	7.7173	7.3187	7.0756	7.0353	7.2977	4.0481	7.6695	2.7568	2.6747
36	7.7157	7.3191	7.0762	7.0359	7.2963	4.0508	7.6732	2.7583	2.6759
40	7.7149	7.3197	7.0771	7.0368	7.2957	4.0539	7.6774	2.7608	2.6778
44	7.7132	7.3201	7.0777	7.0375	7.2941	4.0564	7.6809	2.7623	2.6792
48	7.7117	7.3205	7.0783	7.0382	7.2927	4.059	7.6844	2.7638	2.6808
52	7.7104	7.321	7.079	7.039	7.2917	4.0617	7.6882	2.7665	2.6824
56	7.7095	7.3213	7.0795	7.0395	7.2908	4.0638	7.6909	2.7668	2.6833

Supplementary Table 3 Chemical shifts of different H atoms of BM-HEH under different temperature .

°C/ppm	Ha	Hb	Hb'	Hc	Hd	He	Hh	Hf	Hg1	Hg2
20	7.8277	7.3579	7.2013	7.0542	6.9813	7.2957	4.0316	7.6533	2.803	2.7444
24	7.8286	7.3587	7.204	7.0582	6.9852	7.2996	4.0378	7.6594	2.8069	2.7493
28	7.8286	7.3589	7.2058	7.0608	6.9879	7.3022	4.0417	7.6634	2.8096	2.7522
32	7.8288	7.3587	7.2074	7.0633	6.9904	7.3046	4.0461	7.6681	2.8126	2.7555
36	7.8287	7.3585	7.2088	7.0655	6.9927	7.3068	4.0497	7.6721	2.8146	2.7579
40	7.8281	7.3578	7.21	7.0672	6.9946	7.3085	4.0531	7.6759	2.8166	2.7602
44	7.8274	7.357	7.2111	7.0688	6.9965	7.3101	4.0564	7.6797	2.8186	2.7624
48	7.8269	7.3565	7.2121	7.0705	6.9984	7.3116	4.0596	7.6836	2.8203	2.7646
52	7.8266	7.3561	7.213	7.072	7.0001	7.3131	4.0629	7.6875	2.8222	2.7667
56	7.8263	7.3558	7.2136	7.073	7.0013	7.314	4.0653	7.6904	2.8234	2.768

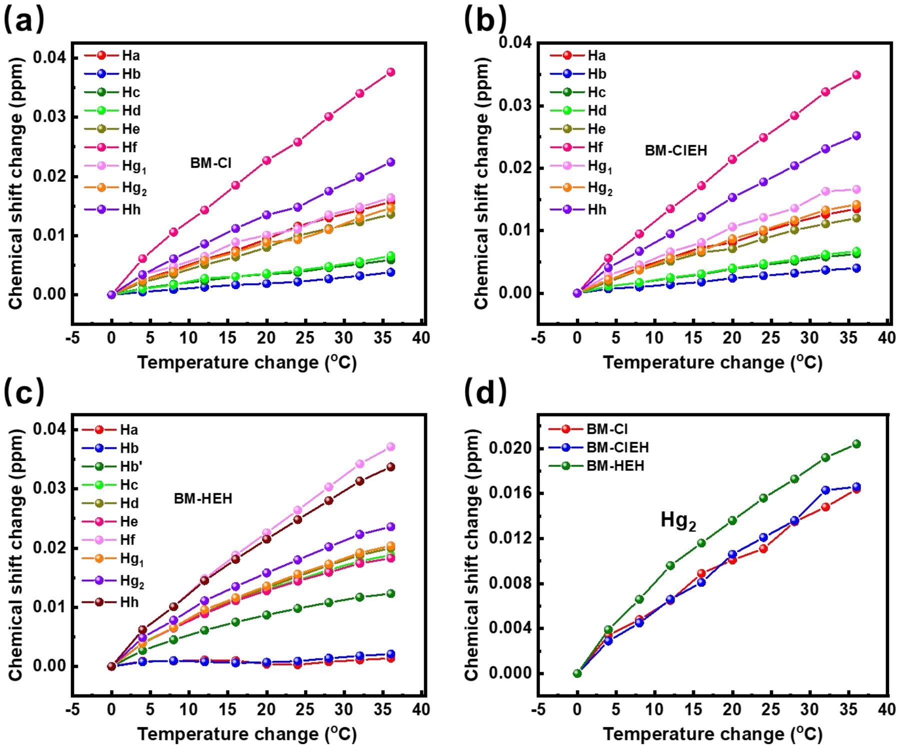

Supplementary Figure 6 Chemical shift changes of different H atoms *vs* temperature changes for (a) BM-Cl; (b) BM-ClEH and (c) BM-HEH. (d) Chemical shift changes of Hg_2 *vs* temperature changes.

GIWAXS measurements for neat films

The GIWAXS characterization were performed at BSRF-1W1A beamline. The samples were prepared under the same conditions with active layer on the Si substrates. The wavelength of incident X-ray was 0.124 nm and the exposure time of samples was 60 s. The incidence light angle of X ray was 0.12° and the scattering signal was collected by mar165CCD with a pixel size of 0.172 mm by 0.172 mm. The sample-to-detector distance was ≈ 252 mm (calibrated by AgB sample). The GIWAXS characterization was done in air environment.

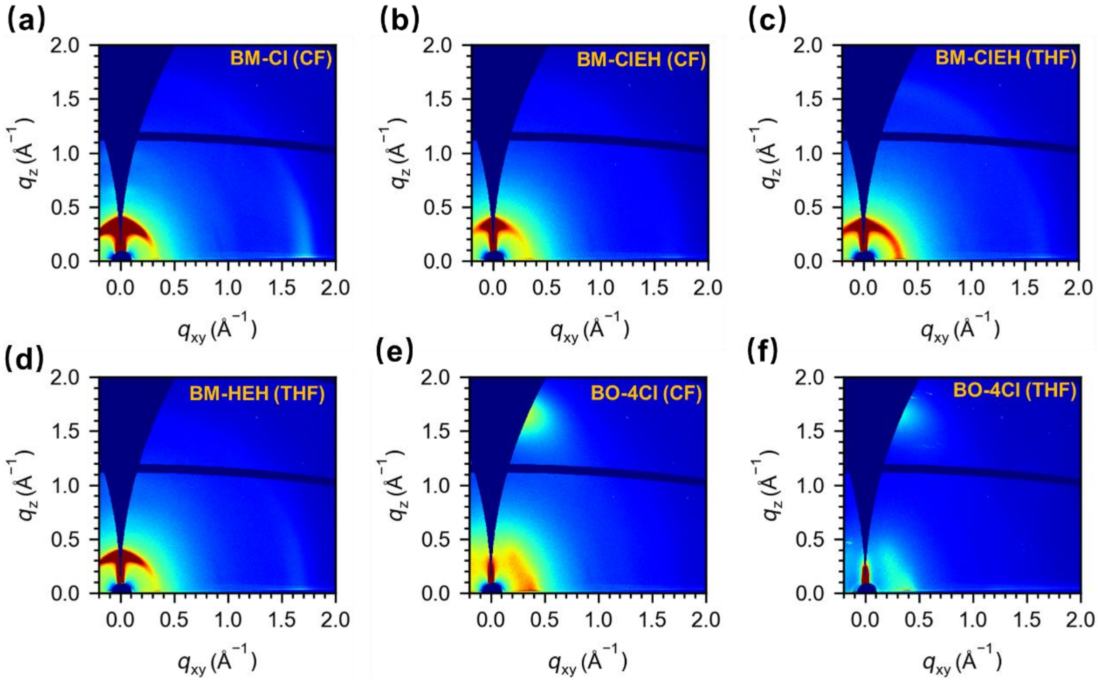

Supplementary Figure 7 2D-GIWAXS patterns of neat films for (a) BM-Cl (CF); (b) BM-ClEH (CF); (c) BM-ClEH (THF); (d) BM-HEH (THF); (e) BO-4Cl (CF) and (f) BO-4Cl (THF).

Supplementary Table 4 Data related to *d*-spacings and coherence lengths were extracted from GIWAXS cut-line profiles.

Samples	(100)				(010)			
	location (Å^{-1})	*d*-spacing (Å)	FWHM (Å^{-1})	CL (Å)	location (Å^{-1})	*d*-spacing (Å)	FWHM (Å^{-1})	CL (Å)
**In-Plane (IP)**								
BM-Cl (CF)	-	-	-	-	1.7175	3.66	0.3787	15.1
BM-ClEH (CF)	-	-	-	-	1.625	3.87	1.3055	4.3
BM-ClEH (THF)	-	-	-	-	1.6325	3.85	1.0258	5.5
BM-HEH (THF)	-	-	-	-	1.6275	3.86	0.7464	7.6
BO-4Cl (CF)	0.365	17.2	1.120	5.0	-	-	-	-
BO-4Cl (THF)	0.385	16.3	0.505	11.2	-	-	-	-
**Out-of-Plane (OOP)**								
BM-Cl (CF)	0.3225	19.5	0.0567	99.8	-	-	-	-
BM-ClEH (CF)	0.3250	19.3	0.0842	67.2	-	-	-	-
BM-ClEH (THF)	0.3225	19.5	0.0710	79.7	-	-	-	-
BM-HEH (THF)	0.3375	18.6	0.0579	97.7	-	-	-	-
BO-4Cl (CF)	-	-	-	-	1.6125	3.90	0.5690	9.9
BO-4Cl (THF)	-	-	-	-	1.615	3.89	0.6255	9.0

SCLC measurements of neat films

The structure of electron-only devices is ITO/ZnO/active layer/PFN-Br:MA/Ag and the structure of hole-only devices is ITO/PEDOT:PSS/active layers/MoOx/Ag. The fabrication conditions of active layer films are same with those for ASM-OSCs. The charge mobilities are generally described by the Mott-Gurney equation:

$$J = \frac{9}{8}\varepsilon_r\varepsilon_0\mu\frac{V^2}{L^3} \quad (1)$$

where J is the current density, ε_0 is the permittivity of free space (8.85×10^{-14} F/cm), ε_r is the dielectric constant of used materials, μ is the charge mobility, V is the applied voltage and L is the active layer thickness. The ε_r parameter is assumed to be 3, which is a typical value for organic materials.

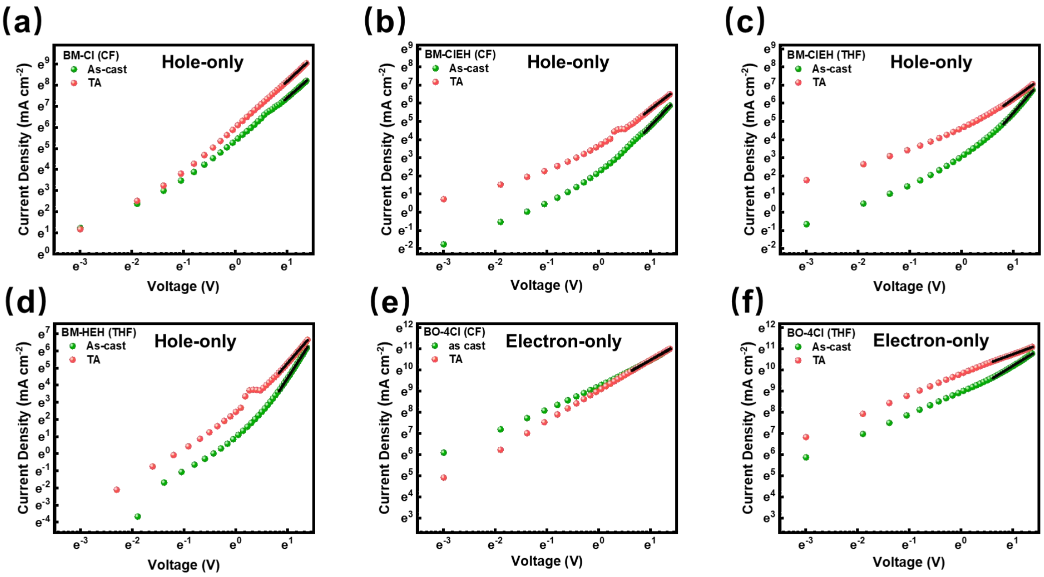

Supplementary Figure 8 *J-V* curves of hole-only SCLC measurements of neat films for (a) BM-Cl (CF); (b) BM-ClEH (CF); (c) BM-ClEH (THF) and (d) BM-HEH (THF). *J-V* curves of electron-only SCLC measurements of neat films for (e) BO-4Cl (CF) and (f) BO-4Cl (THF).

Supplementary Table 5 Hole mobilities of BM-Cl (CF), BM-ClEH (CF), BM-ClEH (THF) and BM-HEH (THF) as-cast and TA films, and electron mobilities of BO-4Cl (CF) and BO-4Cl (THF) as-cast and TA films.

Samples	Hole-mobility (μ_h) (10^{-5} cm^2 V s^{-1})		Electron-mobility (μ_e) (10^{-3} cm^2 V s^{-1})	
	As-cast	TA	As-cast	TA
BM-Cl (CF)	6.27	12.0	-	-
BM-ClEH (CF)	0.199	1.07	-	-
BM-ClEH (THF)	0.302	2.16	-	-
BM-HEH (THF)	0.026	0.206	-	-
BO-4Cl (CF)	-	-	3.08	2.85
BO-4Cl (THF)	-	-	1.99	6.35

Temperature-variable UV-vis absorption spectra of neat films

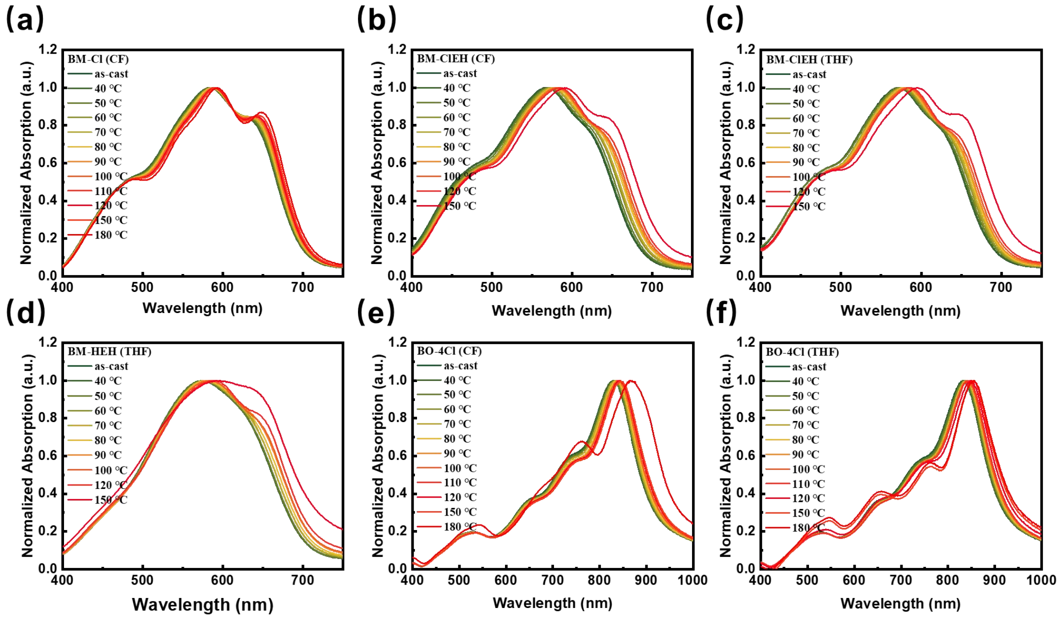

Supplementary Figure 9 Normalized temperature-variable UV-vis absorption spectra of neat films for (a) BM-Cl (CF); (b) BM-ClEH (CF); (c) BM-ClEH (THF); (d) BM-HEH (THF); (e) BO-4Cl (CF) and (f) BO-4Cl (THF) under different thermal annealing temperature.

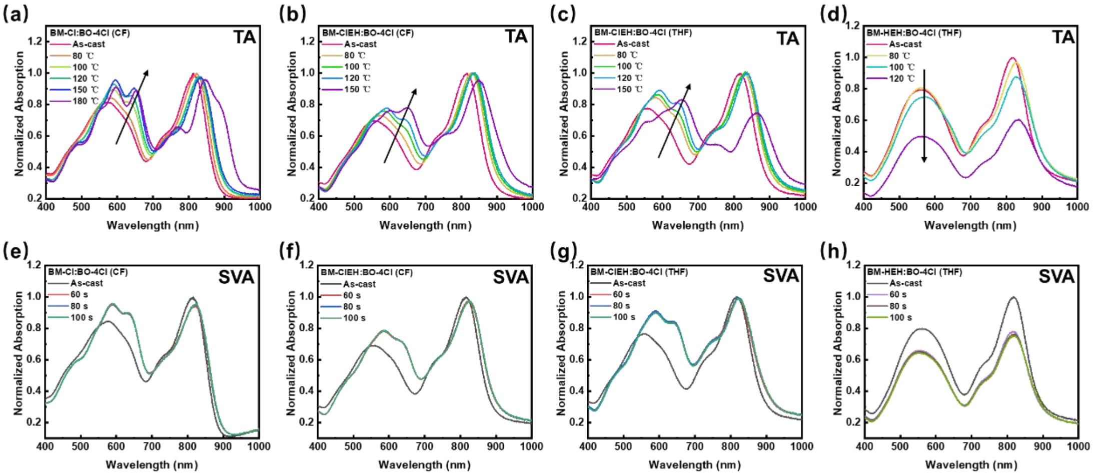

Supplementary Figure 10 Normalized UV-vis absorption spectra of blend films under different thermal annealing temperature: (a) BM-Cl:BO-4Cl (CF); (b) BM-ClEH:BO-4Cl (CF); (c) BM-ClEH:BO-4Cl (THF) and (d) BM-HEH:BO-4Cl (THF). Normalized UV-vis absorption spectra of blend films under different SVA time: (e) BM-Cl:BO-4Cl (CF); (f) BM-ClEH:BO-4Cl (CF); (g) BM-ClEH:BO-4Cl (THF) and (h) BM-HEH:BO-4Cl (THF).

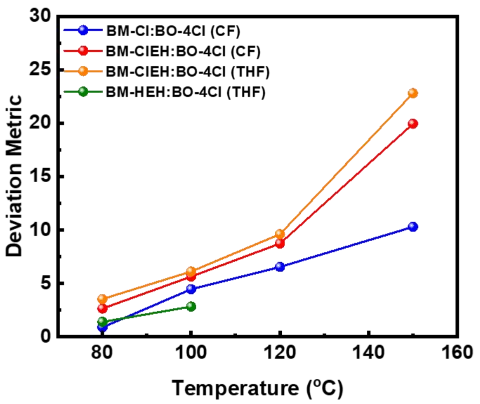

Supplementary Figure 11 Deviation metric of red-shifts measured from temperature-variable UV-vis absorption spectra *vs* temperature for blend films.

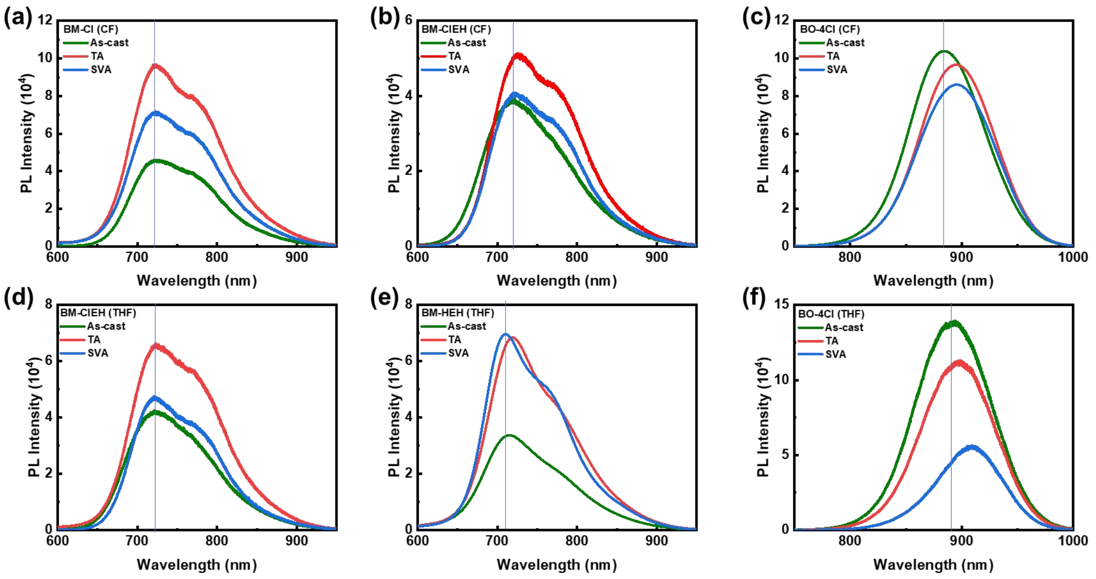

Supplementary Figure 12 PL spectra of neat films for (a) BM-Cl (CF); (b) BM-ClEH (CF); (c) BO-4Cl (CF); (d) BM-ClEH (THF); (e) BM-HEH (THF) and (f) BO-4Cl (THF) without/with TA or SVA treatments.

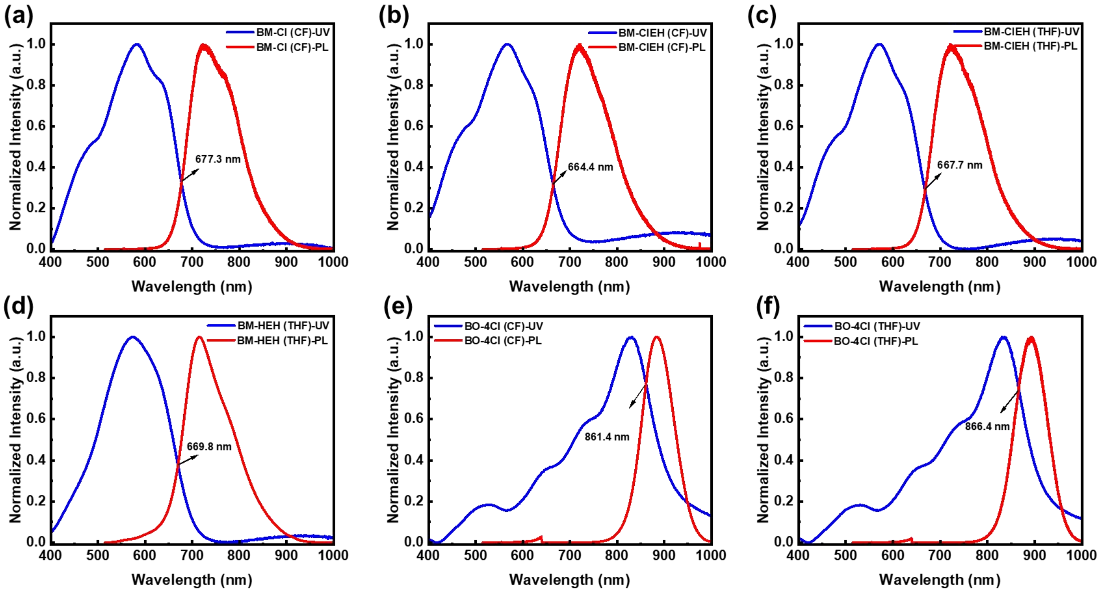

Supplementary Figure 13 Intersections of UV-vis absorption spectra and PL spectra for (a) BM-Cl (CF); (b) BM-ClEH (CF); (c) BM-ClEH (THF); (d) BM-HEH (THF); (e) BO-4Cl (CF) and (f) BO-4Cl (THF) neat films.

UPS measurements

Ultraviolet Photoelectron Spectroscopy (UPS) was performed by PHI 5000

VersaProbe III with He I source (21.22 eV) under an applied negative bias of 10.0 V.

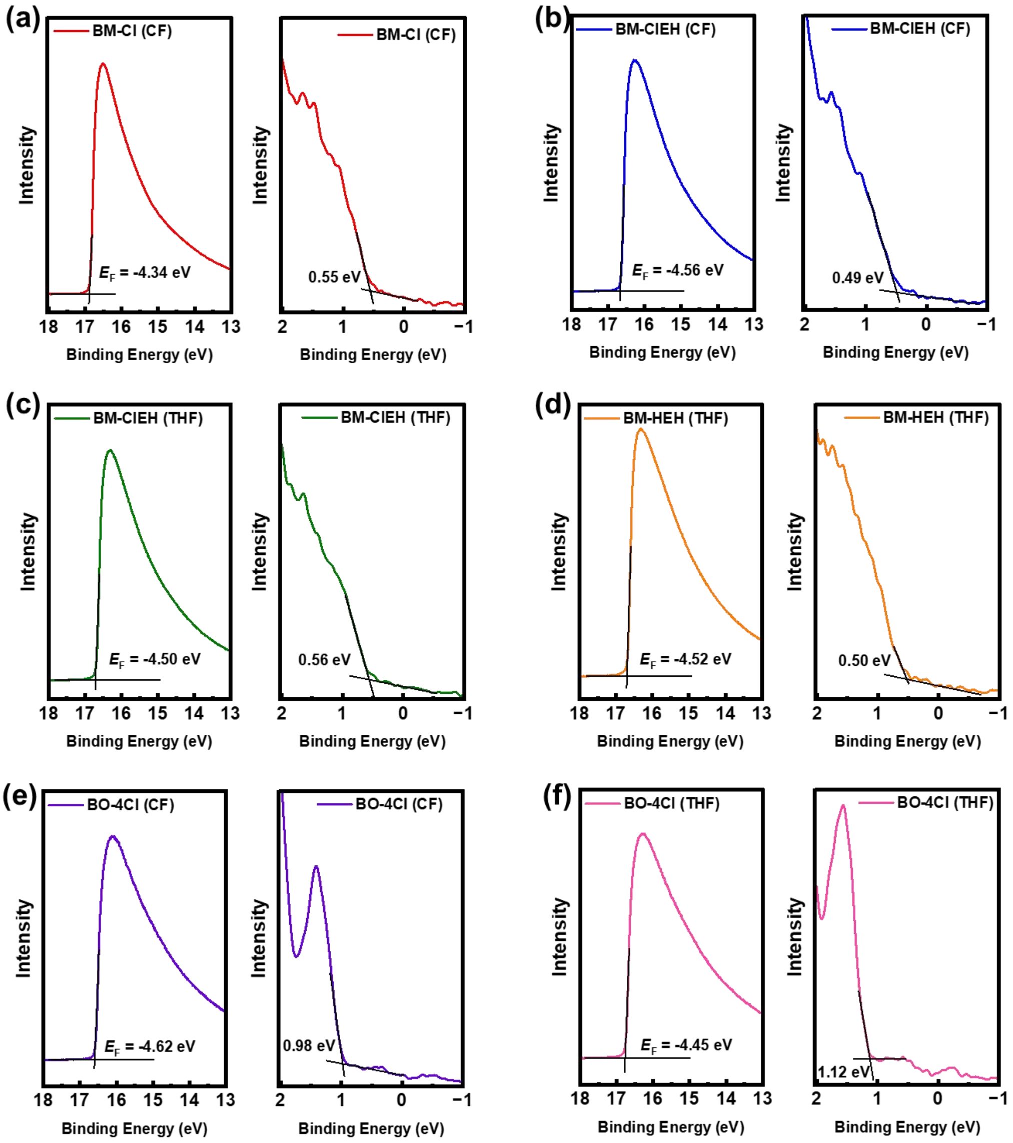

Supplementary Figure 14 UPS tests of neat films for (a) BM-Cl (CF); (b) BM-ClEH (CF); (c) BM-ClEH (THF); (d) BM-HEH (THF); (e) BO-4Cl (CF) and (f) BO-4Cl (THF).

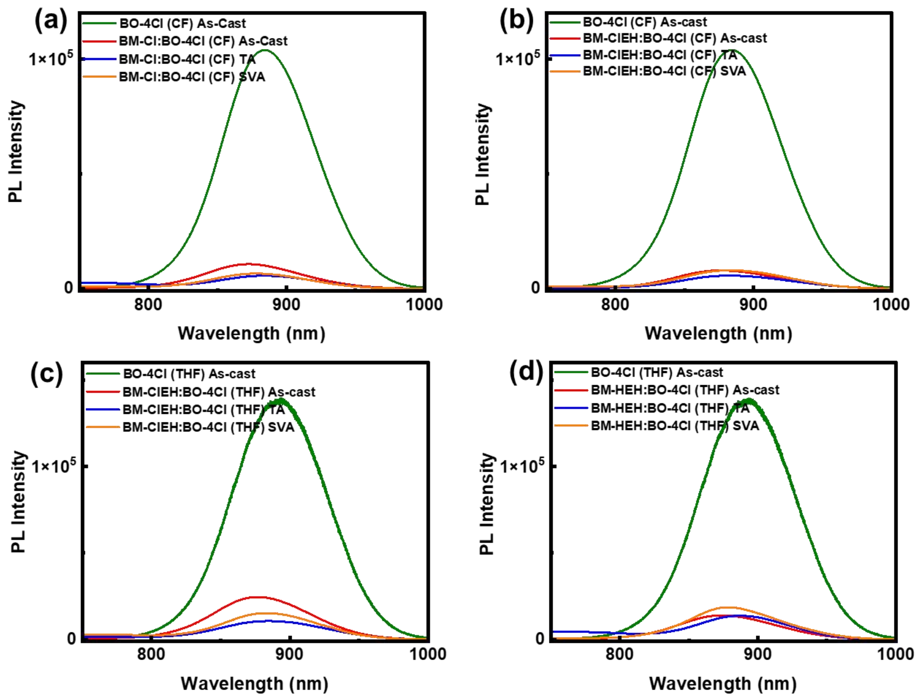

Supplementary Figure 15 PL quenching for (a) BM-Cl:BO-4Cl (CF); (b) BM-ClEH:BO-4Cl (CF); (c) BM-ClEH:BO-4Cl (THF) and (d) BM-HEH:BO-4Cl (THF) blend films.

ASM-OSCs fabrication and characterization

ASM-OSCs were fabricated by using a conventional device structure of ITO/PEDOT:PSS/active layers/PFN-Br:MA/Ag. The patterned ITO-coated glass substrates were first scrubbed by detergent and then cleaned inside an ultrasonic bath by using deionized water, acetone and isopropanol subsequently, and dried overnight in an oven. The glass substrates were treated by UV-Ozone for 30 min before use to improve its work function and clearance. PEDOT:PSS (Al4083 from Hareus) was spin-casted onto the ITO substrates at 7500 rpm for 30 s, and then dried at 160 °C for 15 min in N2 atmosphere. The fully dissolved blend solution of BM-Cl:BO-4Cl in CF, BM-ClEH:BO-4Cl in CF, BM-ClEH:BO-4Cl in THF and BM-HEH:BO-4Cl in THF (weight ratio of 1:1 and total concentration of 20 mg/mL) was spin-casted at 2000 rpm for 30 s onto PEDOT:PSS film followed by a thermal annealing (80-150°C) or solvent evaporation annealing (toluene, 60-100 s). A thin PFN-Br:MA layer (0.5 mg/mL in methanol and 0.25% wt% melamine, 3000 rpm) was coated on the active

layer, followed by the deposition of Ag (evaporated under 3×10^{-4} Pa through a shadow mask). The fabrication process of ternary devices is same as the binary devices by replacing the blend solution with BM-ClEH:B1:BO-4Cl (donor:acceptor weight ratio of 1:1 with B1 ratio of 0, 5, 10, 20, 40 and 100 wt% in donor, total concentration of 20 mg/mL). The optimal active layer thickness measured by a Bruker Dektak XT stylus profilometer was about 110 nm. The current density-voltage (*J-V*) curves of devices were measured using a Keysight B2901A Source Meter in glove box under AM 1.5G (100 mW cm^{-2}) using an Enlitech solar simulator. The device contact area was 0.042 cm^2, device illuminated area during testing was 0.0324 cm^2, which was determined by a mask. The EQE spectra were measured using a Solar Cell Spectral Response Measurement System QE-R3011 (Enlitech Co., Ltd.). The light intensity at each wavelength was calibrated using a standard monocrystalline Si photovoltaic cell. The MPP tracking was carried out upon Epoxy encapsulated devices under 1-sun white LED array in air. The whole tracking condition (temperature and humidity) is co-controlled by air-conditioner and blowing cooling setups.

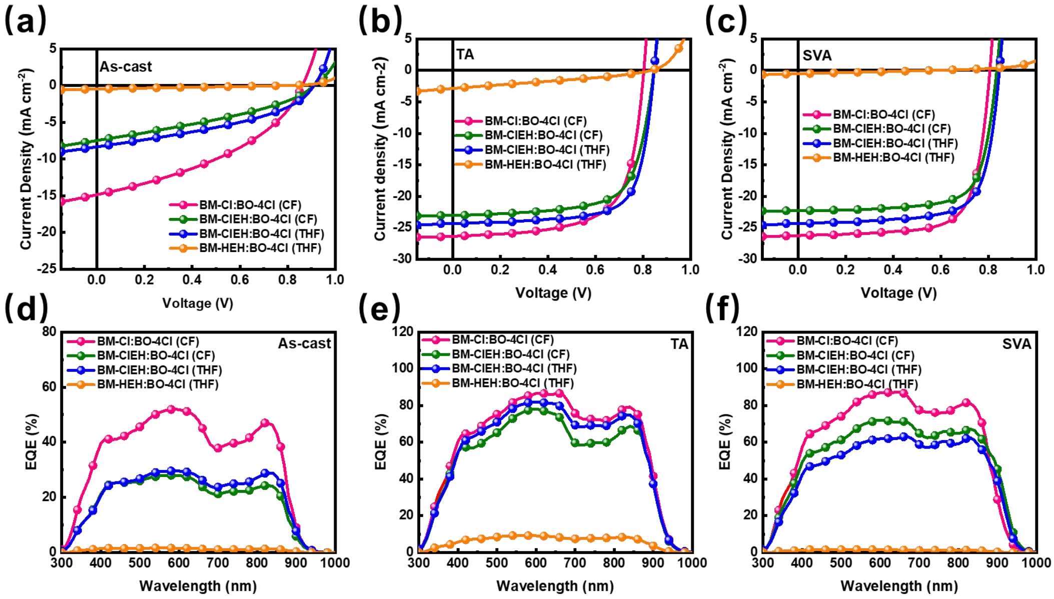

Supplementary Figure 16 Optimal *J-V* curves of ASM-OSCs based on BM-Cl:BO-4Cl (CF), BM-ClEH:BO-4Cl (CF), BM-ClEH:BO-4Cl (THF), BM-HEH:BO-4Cl (THF) (a) without treatments (as-cast); (b) under TA and (c) SVA treatments. Corresponding EQE spectra of ASM-OSCs based on BM-Cl:BO-4Cl (CF),

BM-ClEH:BO-4Cl (CF), BM-ClEH:BO-4Cl (THF), BM-HEH:BO-4Cl (THF) under (d) as-cast; (e) TA and (f) SVA treatments.

Supplementary Table 6 Photovoltaic performance of ASM-OSCs based on BM-Cl:BO-4Cl (CF) under different optimization conditions.

Treatment	V_{oc} (V)	J_{sc} (mA cm^{-2})	J_{sc} (mA cm^{-2})	FF (%)	PCE (%)
As-cast	0.862	14.84	14.40	39.8	5.1
TA (80°C)	0.847	20.91	20.32	47.5	8.4
TA (100°C)	0.821	23.17	/	47.6	9.0
TA (120°C)	0.806	25.60	24.76	61.1	12.6
TA (150°C)	0.803	26.34	25.72	67.4	14.3
SVA (60 s)	0.808	24.86	/	53.5	10.7
SVA (80 s)	0.806	26.23	25.62	72.9	15.4
SVA (100 s)	0.796	23.21	/	72.4	13.4

Supplementary Table 7 Photovoltaic performance of ASM-OSCs based on BM-ClEH:BO-4Cl (CF) under different optimization conditions.

Treatment	V_{oc} (V)	J_{sc} (mA cm^{-2})	J_{sc} (mA cm^{-2})	FF (%)	PCE (%)
As-cast	0.909	7.47	7.93	34.8	2.4
TA (80°C)	0.883	17.81	17.22	46.4	7.3
TA (100°C)	0.859	23.01	22.21	52.8	10.5
TA (120°C)	0.845	23.03	22.62	69.8	13.6
TA (150°C)	0.548	10.85	/	37.8	2.3
SVA (60 s)	0.841	20.89	21.21	75.4	13.2
SVA (80 s)	0.835	22.26	21.95	73.6	13.7
SVA (100 s)	0.833	21.62	21.45	70.8	12.8

Supplementary Table 8 Photovoltaic performance of ASM-OSCs based on BM-ClEH:BO-4Cl (THF) under different optimization conditions.

Treatment	V_{oc} (V)	J_{sc} (mA cm^{-2})	J_{sc} (mA cm^{-2})	FF (%)	PCE (%)
As-cast	0.910	8.32	8.66	39.6	3.0
TA (80°C)	0.870	21.48	20.65	66.4	12.4
TA (100°C)	0.846	24.38	24.22	72.7	15.0
TA (120°C)	0.811	20.04	20.48	73.7	12.0
TA (150°C)	/	/	/	/	/
SVA (60 s)	0.838	19.16	/	75.3	12.1
SVA (80 s)	0.838	20.36	19.53	74.4	12.7
SVA (100 s)	0.836	20.39	/	72.9	12.4

Supplementary Table 9 Photovoltaic performance of ASM-OSCs based on BM-HEH:BO-4Cl (THF) under different optimization conditions.

Treatment	V_{oc} (V)	J_{sc} (mA cm^{-2})	J_{sc} (mA cm^{-2})	FF (%)	PCE (%)
As-cast	0.795	0.47	0.49	24.9	0.1
TA (100°C)	0.834	2.87	2.68	30.5	0.7
SVA (80 s)	0.574	0.54	0.45	26.1	0.1

Supplementary Table 10 Photovoltaic performance of reported ASM-OSCs based on green solvent processing.

Active layer	Processing solvent	V_{oc} (V)	J_{sc} (mA cm^{-2})	FF (%)	PCE (%)	Year	Ref.
BTR:$PC_{71}BM$	Toluene	0.91	11.2	72.3	7.46	2017	4
N(Ph-2T-DCN-Et)$_3$:$PC_{71}BM$	benzaldehyde	0.96	8.27	46.75	3.71	2014	5
DPP-EZnP-O:$PC_{61}BM$	o-Xylene	0.75	15.73	50.0	5.85	2015	6
DRTT-R:F-2Cl	THF	1.00	16.82	62.6	10.45	2019	7
ZR1-C3:L8-BO	THF	0.897	23.24	67.41	14.05	2023	8

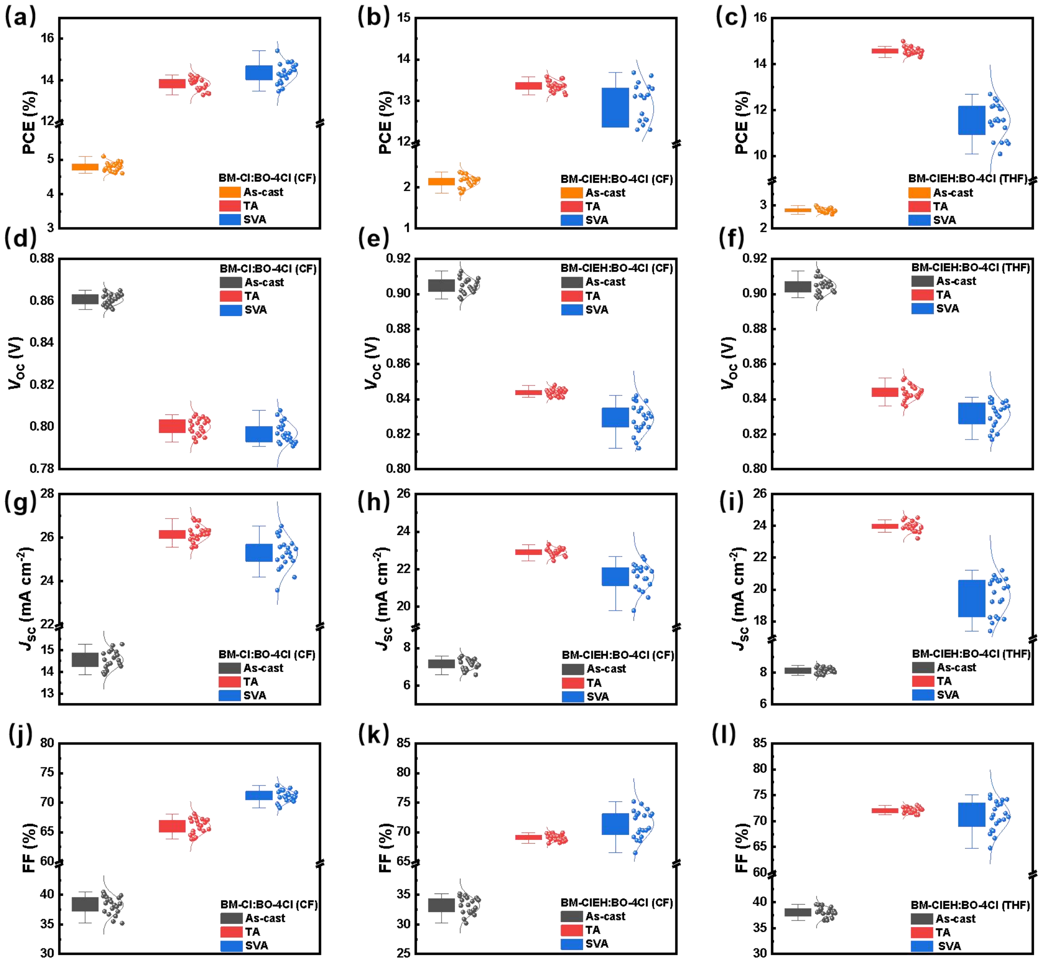

Supplementary Figure 17 Photovoltaic parameters repeatability of ASM-OSCs based on BM-Cl:BO-4Cl (CF), BM-ClEH:BO-4Cl (CF) and BM-ClEH:BO-4Cl: (a-c) PCE; (d-f) V_{OC}; (g-i) J_{SC} and (j-l) FF.

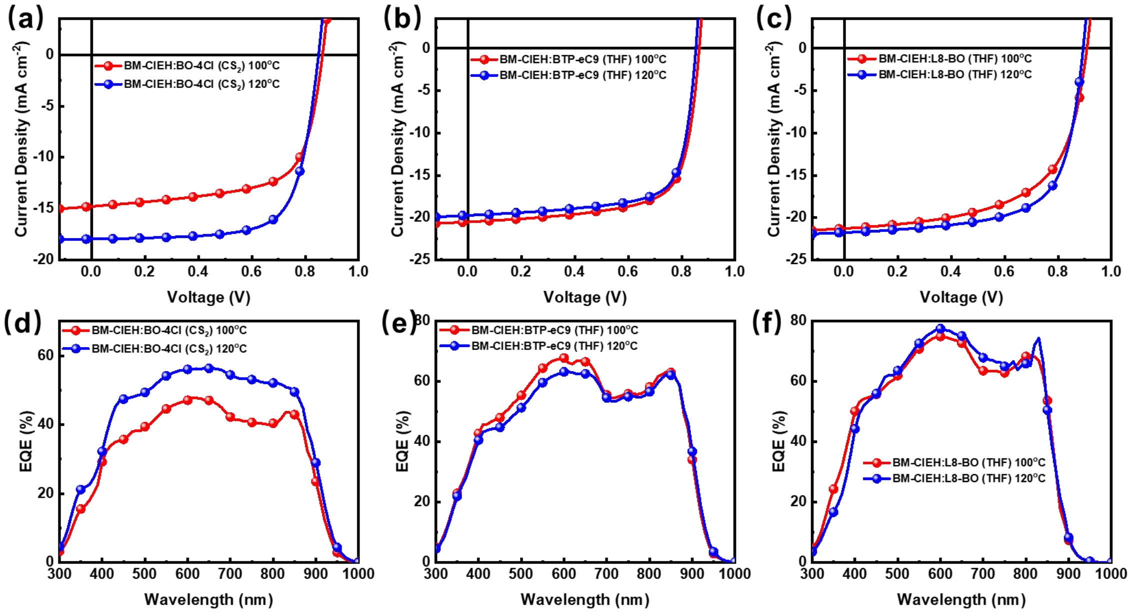

Supplementary Figure 18 *J-V* curves for ASM-OSCs based on (a) BM-ClEH:BO-4Cl fabricated with CS_2; (b) BM-ClEH:BTP-eC9 (THF) and (c) BM-ClEH:L8-BO (THF). EQE spectra for ASM-OSCs based on (d) BM-ClEH:BO-4Cl fabricated with CS_2; (e) BM-ClEH:BTP-eC9 (THF) and (f) BM-ClEH:L8-BO (THF).

Supplementary Table 11 Photovoltaic parameters for ASM-OSCs based on BM-ClEH:BO-4Cl fabricated with CS_2, BM-ClEH:BTP-eC9 (THF) and BM-ClEH:L8-BO (THF).

Active Layer	V_{oc} (V)	J_{sc} (mA cm^{-2})	$J_{sc}$$^{a)}$ (mA cm^{-2})	FF (%)	PCE (%)
BM-ClEH:BO-4Cl (CS_2, 100°C)	0.865	14.78	13.98	66.5	8.5
BM-ClEH:BO-4Cl (CS_2, 120°C)	0.850	17.95	17.29	71.2	10.9
BM-ClEH:BTP-eC9 (THF, 100°C)	0.866	20.50	19.71	70.7	12.6
BM-ClEH:BTP-eC9 (THF, 120°C)	0.853	19.72	18.95	72.6	12.2
BM-ClEH:L8-BO (THF, 100°C)	0.908	21.27	20.65	60.3	11.7
BM-ClEH:L8-BO (THF, 120°C)	0.894	21.75	21.02	67.1	13.1

[a]Integated from EQE spectra.

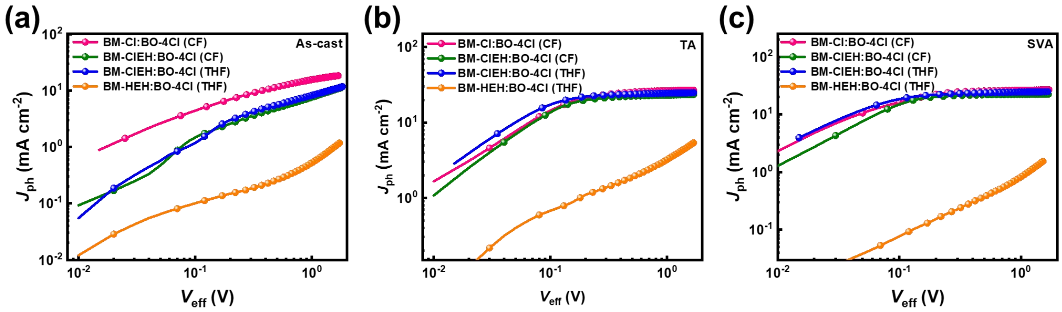

Supplementary Figure 19 J_{ph} *vs* V_{eff} plots of (a) as-cast, (b) TA or (c) SVA treated devices based on BM-Cl:BO-4Cl (CF), BM-ClEH:BO-4Cl (CF), BM-ClEH:BO-4Cl (THF) and BM-HEH:BO-4Cl.

Supplementary Table 12 Exciton dissociation and charge collection efficiencies extracted from J_{ph} *vs* V_{eff} plots.

Active layer	J_{sat} (mA cm^{-2})	J_{ph}^{*} (mA cm^{-2})	$J_{ph}^{\&}$ (mA cm^{-2})	J_{ph}^{*}/J_{sat} (%)	$J_{ph}^{\&}/J_{sat}$ (%)
**TA**					
BM-Cl:BO-4Cl (CF)	26.89	26.34	21.94	98.0	81.6
BM-ClEH:BO-4Cl (CF)	23.54	23.03	19.70	97.8	83.7
BM-ClEH:BO-4Cl (THF)	24.86	24.38	21.06	97.8	84.7
BM-HEH:BO-4Cl (THF)	5.37	2.87	1.43	53.4	49.8
**SVA**					
BM-Cl:BO-4Cl (CF)	26.81	26.23	22.99	97.8	85.7
BM-ClEH:BO-4Cl (CF)	24.88	22.26	21.06	89.5	84.6
BM-ClEH:BO-4Cl (THF)	22.45	20.36	19.54	90.7	87.0
BM-HEH:BO-4Cl (THF)	1.54	0.54	0.27	35.0	17.5

J_{sat}: saturation photocurrent density

J_{ph}^{*}: Photocurrent density under short circuit conditions

$J_{ph}^{\&}$: Photocurrent density under maximum power output conditions

SCLC measurements of blend films

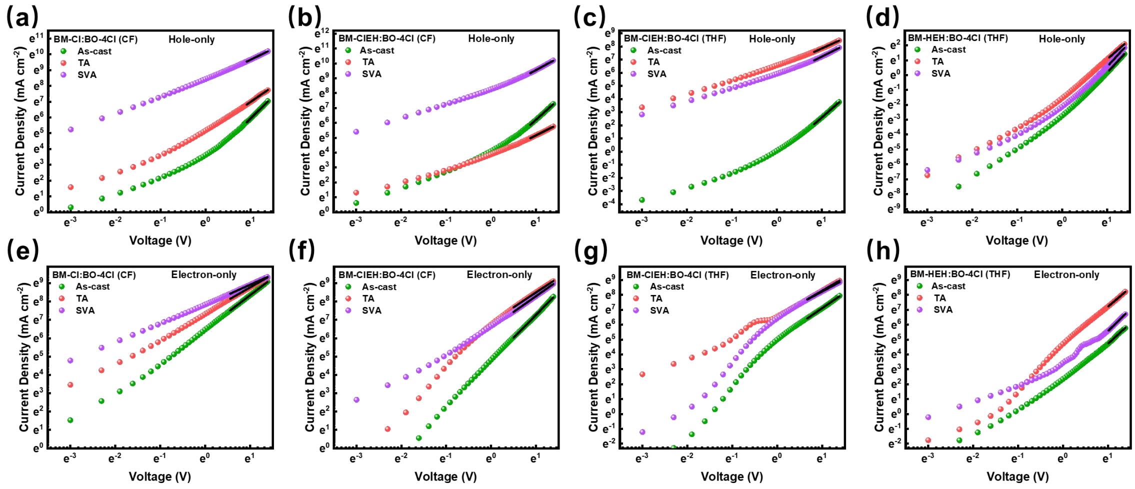

Supplementary Figure 20 *J-V* curves of hole-only SCLC measurements for (a) BM-Cl:BO-4Cl (CF); (b) BM-ClEH:BO-4Cl (CF); (c) BM-ClEH:BO-4Cl (THF) and (d) BM-HEH:BO-4Cl (THF). *J-V* curves of electron-only SCLC measurements for (e) BM-Cl:BO-4Cl (CF); (f) BM-ClEH:BO-4Cl (CF); (g) BM-ClEH:BO-4Cl (THF) and (h) BM-HEH:BO-4Cl (THF).

Supplementary Table 13 Hole and electron mobilities of BM-Cl:BO-4Cl (CF), BM-ClEH:BO-4Cl (CF), BM-ClEH:BO-4Cl (THF) and BM-HEH:BO-4Cl (THF) active layers under different conditions.

Samples	Hole-mobility (μ_h) (10^{-5} cm^2 V s^{-1})			Electron-mobility (μ_e) (10^{-5} cm^2 V s^{-1})			Ratio (μ_e/μ_h)		
	As-cast	TA	SVA	As-cast	TA	SVA	As-Cast	TA	SVA
BM-Cl:BO-4Cl (CF)	0.74	5.01	132.6	21.4	52.9	82.9	28.9	10.6	0.63
BM-ClEH:BO-4Cl (CF)	1.08	1.28	90.6	4.03	32.2	24.0	3.73	25.1	0.26
BM-ClEH:BO-4Cl (THF)	0.02	18.2	8.41	5.13	22.9	24.9	256.5	1.26	2.96
BM-HEH:BO-4Cl (THF)	0.001	0.007	0.002	0.08	0.25	0.10	80	35.7	50

AFM measurements

AFM measurements were obtained by using a Dimension Icon AFM (Bruker) in a tapping mode.

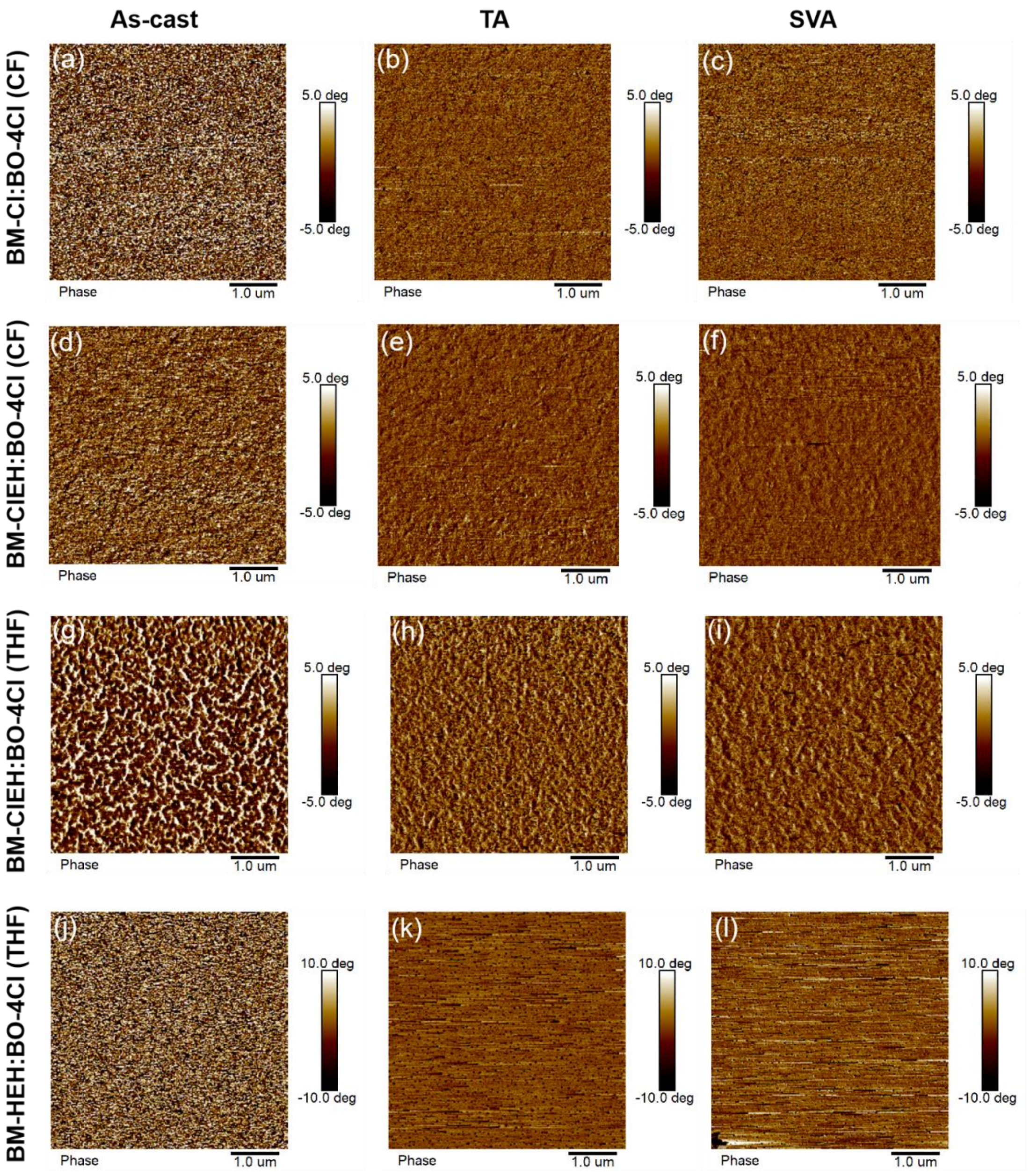

Supplementary Figure 21 AFM phase images: (a-c) BM-Cl:BO-4Cl (CF); (d-f) BM-ClEH:BO-4Cl (CF); (g-i) BM-ClEH:BO-4Cl (THF) and (j-l) BM-HEH:BO-4Cl (THF) active layers under as-cast, TA and SVA conditions, respectively.

GIWAXS measurements of blend films

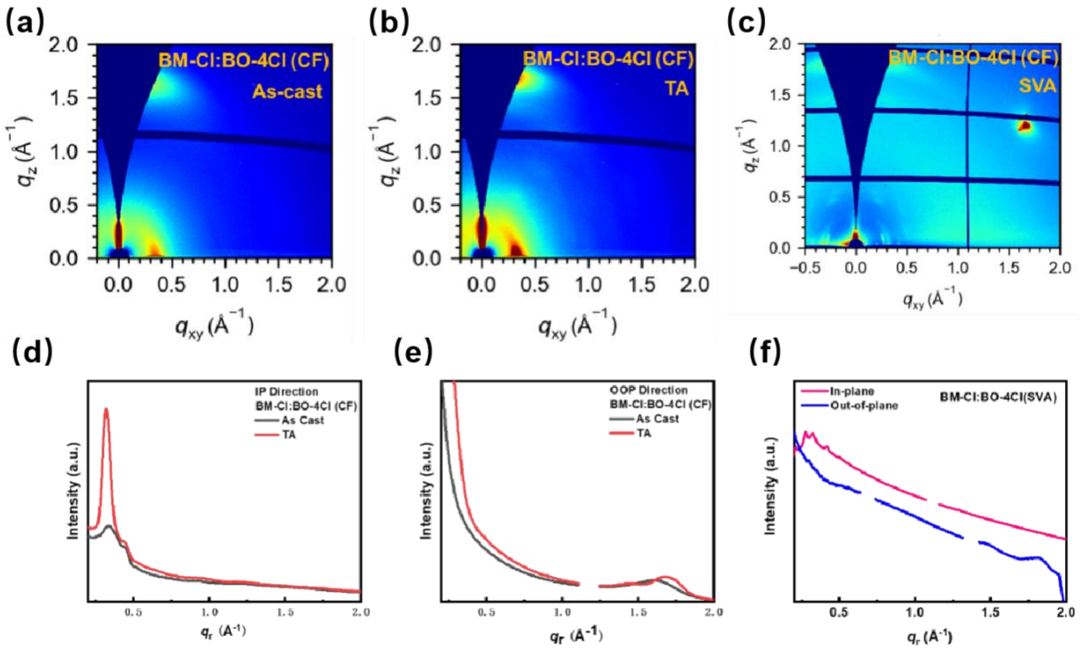

Supplementary Figure 22 2D-GIWAXS patterns of BM-Cl:BO-4Cl (CF) blend film under (a) as-cast; (b) TA and (c) SVA treatments. Corresponding cut-line profiles for (d) as-cast and thermal annealed BM-Cl:BO-4Cl (CF) blend film in IP direction; (e) as-cast and thermal annealed BM-Cl:BO-4Cl (CF) blend film in OOP direction and (f) BM-Cl:BO-4Cl (CF) blend film under SVA treatment in IP and OOP directions.

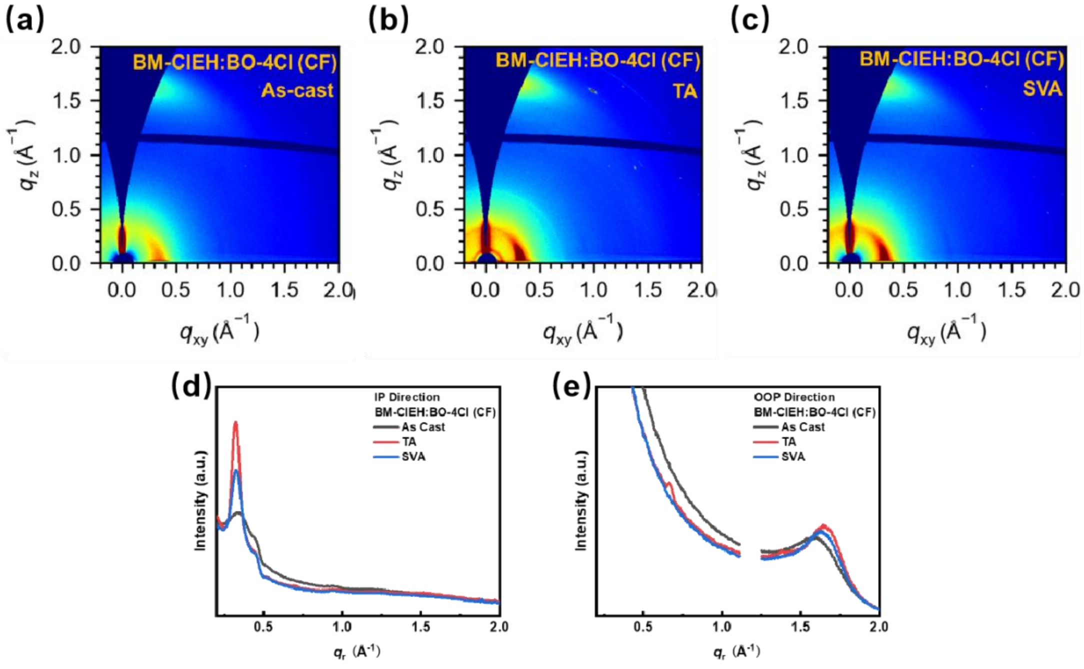

Supplementary Figure 23 2D-GIWAXS patterns of BM-ClEH:BO-4Cl (CF) blend film under (a) as-cast; (b) TA and (c) SVA treatments. Corresponding cut-line profiles

(d) in IP direction and (e) in OOP direction.

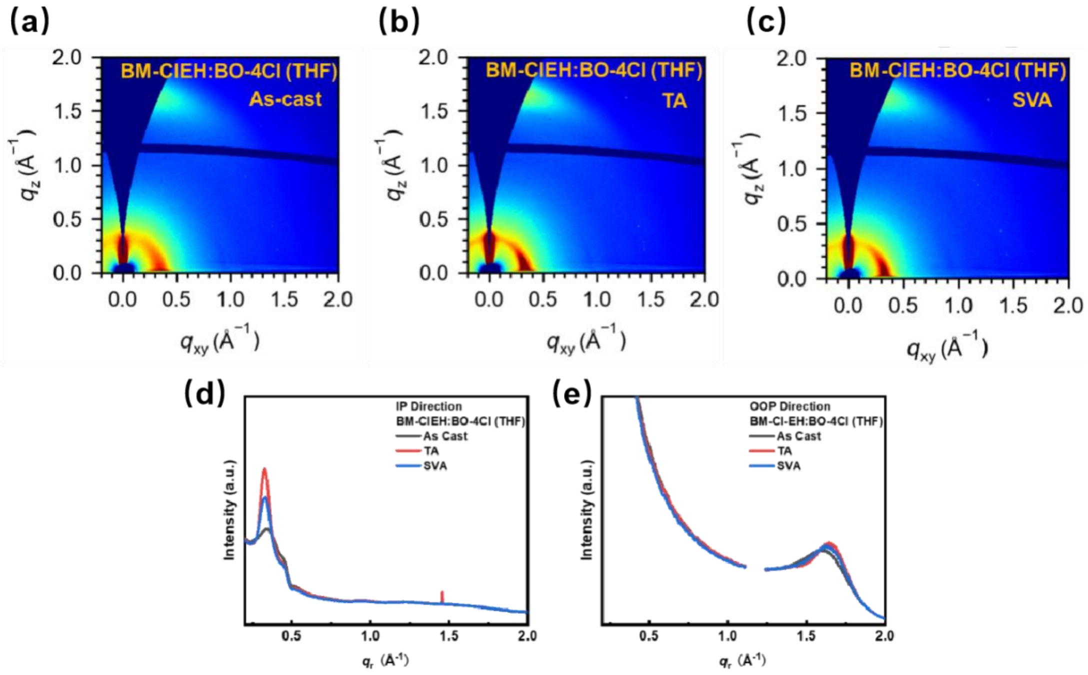

Supplementary Figure 24 2D-GIWAXS patterns of BM-ClEH:BO-4Cl (THF) blend film under (a) as-cast; (b) TA and (c) SVA treatments. Corresponding cut-line profiles (d) in IP direction and (e) in OOP direction.

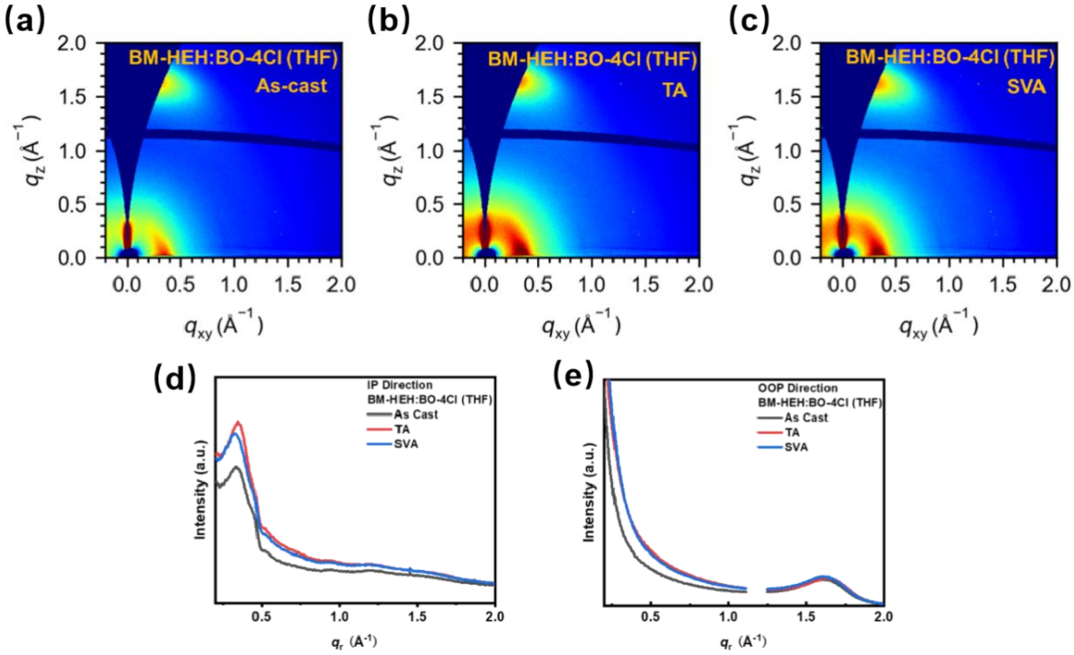

Supplementary Figure 25 2D-GIWAXS patterns of BM-HEH:BO-4Cl (THF) blend film under (a) as-cast; (b) TA and (c) SVA treatments. Corresponding cut-line profiles

(d) in IP direction and (e) in OOP direction.

Supplementary Table 14 Data related to *d*-spacings and coherence lengths were extracted from GIWAXS cut-line profiles.

Samples	(100)				(010)			
	location ($Å^{-1}$)	*d*-spacing (Å)	FWHM ($Å^{-1}$)	CL (Å)	location ($Å^{-1}$)	*d*-spacing (Å)	FWHM ($Å^{-1}$)	CL (Å)
**In-Plane (IP)**								
BM-Cl:BO-4Cl (CF)								
As-cast	0.355	17.7	0.3361	16.8	-	-	-	-
TA	0.320	19.6	0.0843	67.1	-	-	-	-
SVA	0.325	19.3	0.1735	32.6	-	-	-	-
BM-ClEH:BO-4Cl (CF)								
As-cast	0.345	18.2	0.5237	10.8	-	-	-	-
TA	0.323	19.4	0.0901	62.8	-	-	-	-
SVA	0.325	19.3	0.1523	37.1	-	-	-	-
BM-ClEH:BO-4Cl (THF)								
As-cast	0.335	18.7	0.4175	13.5	-	-	-	-
TA	0.327	19.2	0.1212	46.7	-	-	-	-
SVA	0.333	18.8	0.1606	35.2	-	-	-	-
BM-HEH:BO-4Cl (THF)								
As-cast	0.338	18.6	0.4448	12.7	-	-	-	-
TA	0.348	18.0	0.3704	15.3	-	-	-	-
SVA	0.330	19.0	0.3989	14.2	-	-	-	-
**Out-of-Plane (OOP)**								
BM-Cl:BO-4Cl (CF)								
As-cast	-	-	-	-	1.615	3.89	0.5810	9.7
TA	-	-	-	-	1.685	3.73	0.3354	16.9
SVA	-	-	-	-	1.710	3.67	0.4120	13.7
BM-ClEH:BO-4Cl (CF)								
As-cast	-	-	-	-	1.588	3.95	0.8795	6.4
TA	-	-	-	-	1.643	3.82	0.4319	13.1
SVA	-	-	-	-	1.630	3.85	0.5588	10.1
BM-ClEH:BO-4Cl (THF)								
As-cast	-	-	-	-	1.588	3.95	0.7112	7.9
TA	-	-	-	-	1.642	3.82	0.3937	14.4
SVA	-	-	-	-	1.630	3.85	0.5987	9.4
BM-HEH:BO-4Cl (THF)								
As-cast	-	-	-	-	1.603	3.92	0.7583	7.5

TA	-	-	-	-	1.621	3.87	0.7013	8.1
SVA	-	-	-	-	1.603	3.92	0.7351	7.1

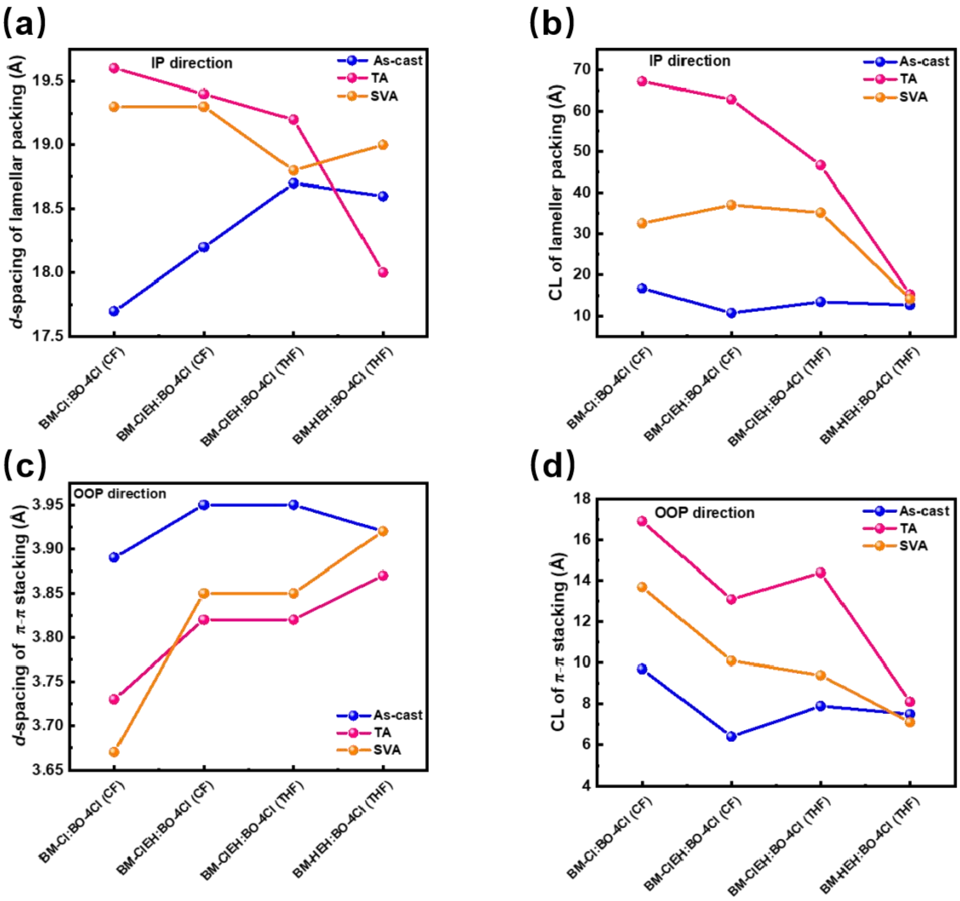

Supplementary Figure 26 (a) *d*-spacings and (b) CLs of lamellar packing for BM-Cl:BO-4Cl (CF), BM-ClEH:BO-4Cl (CF), BM-ClEH:BO-4Cl (THF) and BM-HEH:BO-4Cl (THF) active layer under different conditions. (c) *d*-spacings and (d) CLs of π-π stacking for BM-Cl:BO-4Cl (CF), BM-ClEH:BO-4Cl (CF), BM-ClEH:BO-4Cl (THF) and BM-HEH:BO-4Cl (THF) active layer under different conditions.

GISAXS measurements

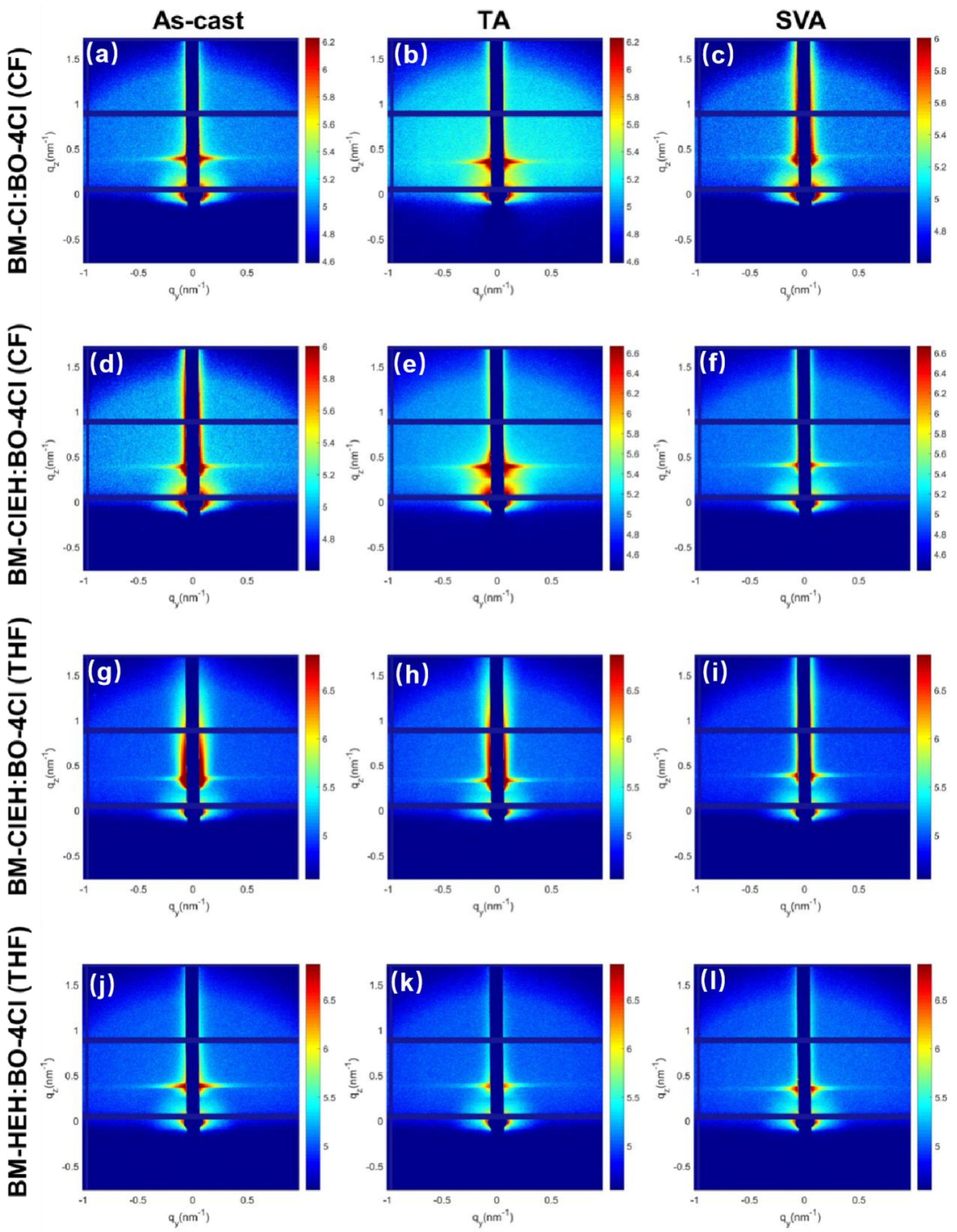

Supplementary Figure 27 2D-GISAXS patterns of BM-Cl:BO-4Cl (CF) blend film under (a) as-cast; (b) TA and (c) SVA treatments. 2D-GISAXS patterns of BM-ClEH:BO-4Cl (CF) blend film under (d) as-cast; (e) TA and (f) SVA treatments. 2D-GISAXS patterns of BM-ClEH:BO-4Cl (THF) blend film under (g) as-cast; (h) TA and (i) SVA treatments. 2D-GISAXS patterns of BM-HEH:BO-4Cl (THF) blend film under (j) as-cast; (k) TA and (l) SVA treatmets.

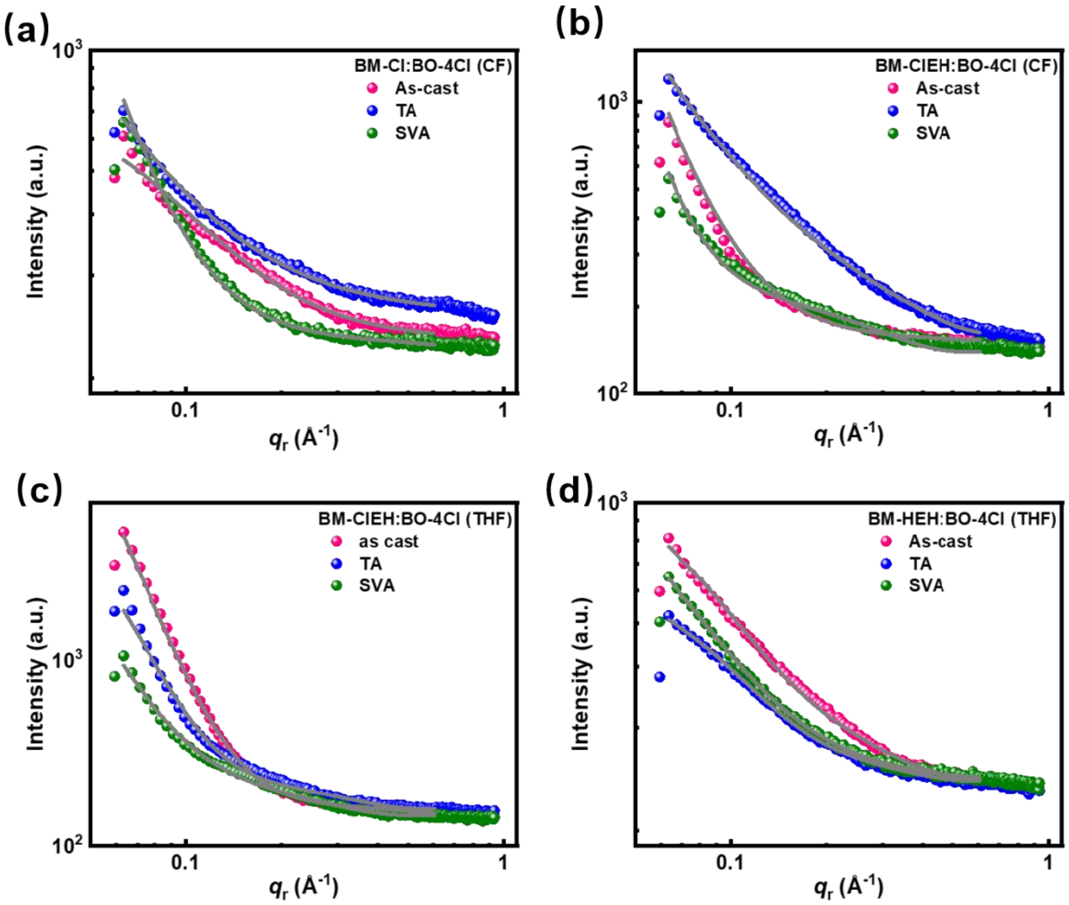

Supplementary Figure 28 Intensity profiles of GISAXS based on (a) BM-Cl:BO-4Cl (CF); (b) BM-ClEH:BO-4Cl (CF); (c) BM-ClEH:BO-4Cl (THF) and (d) BM-HEH:BO-4Cl (THF) active layer under different conditions.

Supplementary Table 15 Data extracted from GISAXS.

Samples	Treatment	ζ (nm)	η (nm)	D	$2R_g$ (nm)
BM-Cl:BO-4Cl (CF)	As-cast	18.56	5.73	2.46	23.64
	TA	19.24	5.96	2.68	26.47
	SVA	18.97	5.82	2.52	24.51
BM-ClEH:BO-4Cl (CF)	As-cast	20.06	5.86	2.59	25.27
	TA	21.12	6.11	2.86	28.71
	SVA	23.79	5.97	2.73	26.94
BM-ClEH:BO-4Cl (THF)	As-Cast	22.31	6.25	3.09	31.42
	TA	24.35	6.63	3.20	32.15
	SVA	25.06	6.41	3.18	34.37
BM-HEH:BO-4Cl (THF)	As-cast	19.67	5.78	2.67	25.59
	TA	20.14	5.82	2.75	26.43
	SVA	20.67	5.71	2.71	25.60

TAS measurements

Transient absorption spectroscopy (TAS) was measured with an amplified Ti:sapphire femtosecond laser (800 nm wavelength, 50 fs, 1 kHz repetition; Coherent Libra) and a Helios pump/probe setup (Ultrafast Systems). The 400 nm pump pulses with a pump fluence of 0.5 or < 0.3 μJ/cm^2 were obtained by frequency doubling the 800 nm fundamental regenerative amplifier output. The white-light continuum probe pulses were generated by focusing a small portion of the regenerative amplifier's fundamental 800 nm laser pulses into a 2 mm sapphire crystal.

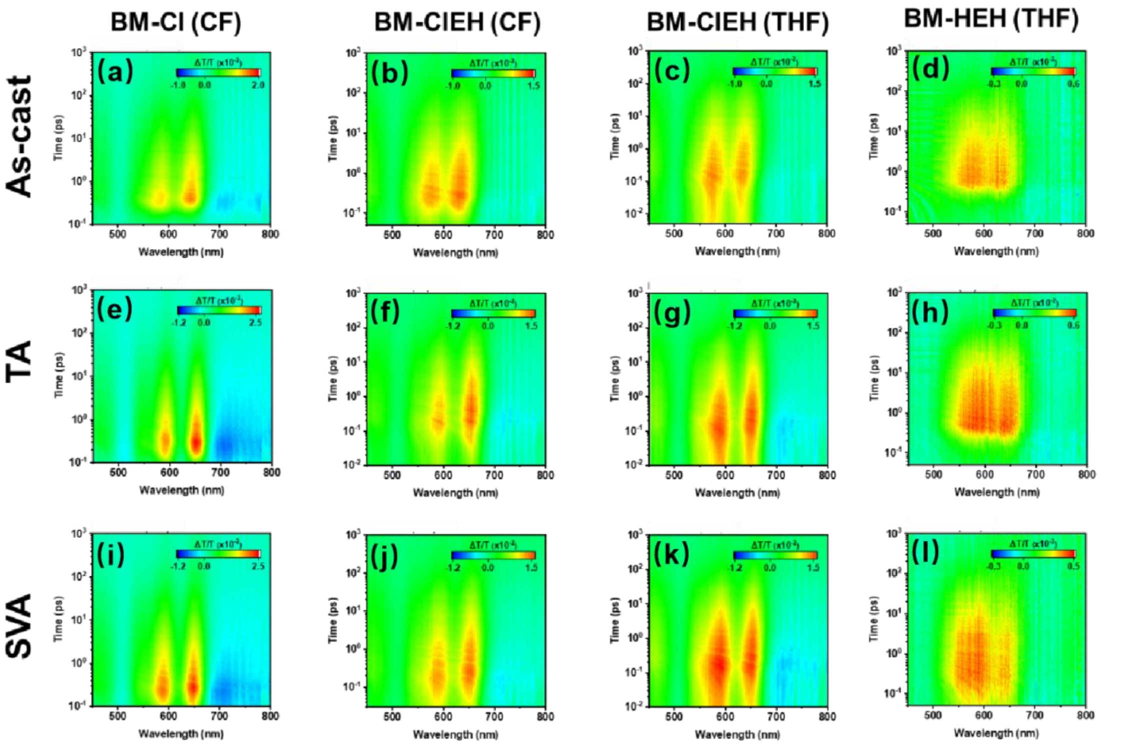

Supplementary Figure 29 2D plots of TAS measurements for (a) BM-Cl (CF); (b) BM-ClEH (CF); (c) BM-ClEH (THF) and (d) BM-HEH (THF) as-cast films. 2D plots of TAS measurements for (e) BM-Cl (CF); (f) BM-ClEH (CF); (g) BM-ClEH (THF) and (h) BM-HEH (THF) TA films. 2D plots of TAS measurements for (i) BM-Cl (CF); (j) BM-ClEH (CF); (k) BM-ClEH (THF) and (l) BM-HEH (THF) SVA films.

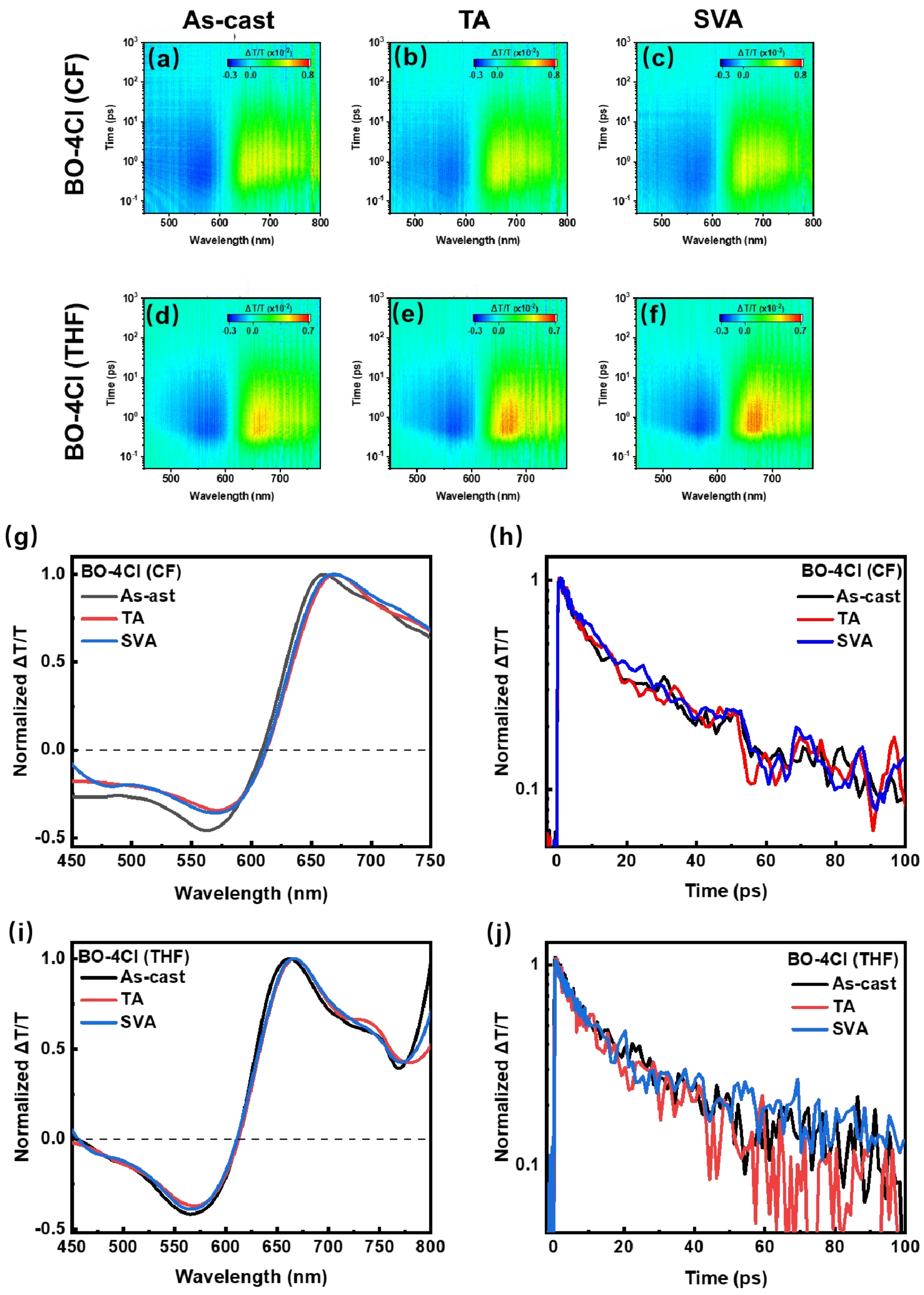

Supplementary Figure 30 2D plots of TAS measurements for BO-4Cl (CF) under (a) as-cast; (b) TA and (c) SVA treatments. 2D plots of TAS measurements for BO-4Cl (THF) under (d) as-cast; (e) TA and (f) SVA treatments. (g) Spectral cuts immediately after excitation (0.5-1 ps) for BO-4Cl (CF) films under different treatments and (h) corresponding TAS kinetics of singlet excitons probed at 650-690 nm. (i) Spectral

cuts immediately after excitation (0.5-1 ps) for BO-4Cl (THF) films under different treatments and (j) corresponding TAS kinetics of singlet excitons probed at 650-690 nm.

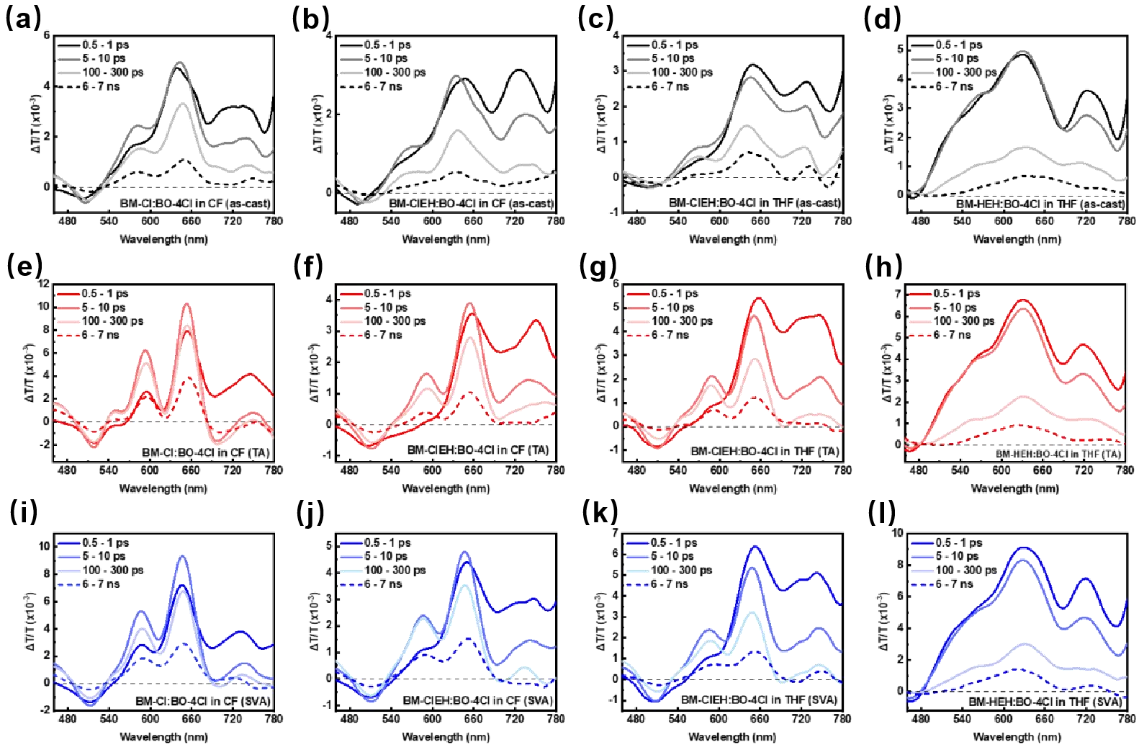

Supplementary Figure 31 Spectral cuts of TAS measurements after different time for as-cast blend films: (a) BM-Cl:BO-4Cl (CF); (b) BM-ClEH:BO-4Cl (CF); (c) BM-ClEH:BO-4Cl (THF) and (d) BM-HEH:BO-4Cl (THF). Spectral cuts of TAS measurements after different time for TA blend films: (e) BM-Cl:BO-4Cl (CF); (f) BM-ClEH:BO-4Cl (CF); (g) BM-ClEH:BO-4Cl (THF) and (h) BM-HEH:BO-4Cl (THF). Spectral cuts of TAS measurements after different time for SVA blend films: (i) BM-Cl:BO-4Cl (CF); (j) BM-ClEH:BO-4Cl (CF); (k) BM-ClEH:BO-4Cl (THF) and (l) BM-HEH:BO-4Cl (THF).

Supplementary Table 16 Photovoltaic parameters of ternary ASM-OSCs based on BM-ClEH:B1:BO-4Cl processed by THF.

B1 weight ratio	V_{oc} (V)	J_{sc} (mA cm^{-2})	J_{sc}^{EQE} (mA cm^{-2})	FF (%)	PCE (%)
0%	0.846	24.32	24.22	72.7	15.0
5%	0.842	25.58	25.08	71.4	15.4
10%	0.837	26.96	26.58	71.2	16.1
20%	0.829	26.15	25.80	66.7	14.5
40%	0.822	25.23	24.70	57.4	11.9
100%	0.814	24.19	23.93	48.2	9.5

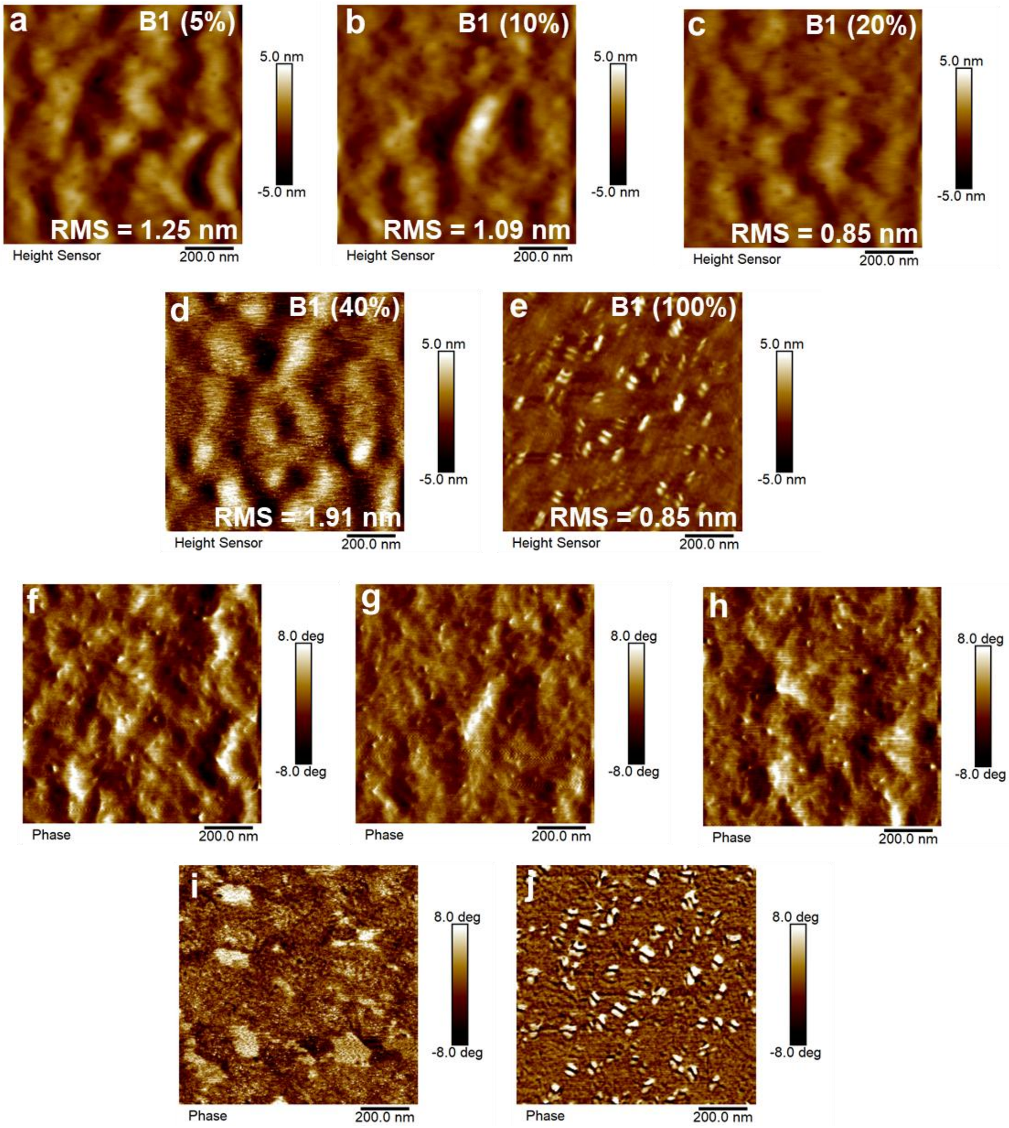

Supplementary Figure 32 AFM height images of BM-ClEH:B1:BO-4Cl blend films with (a) 5%, (b) 10%, (c) 20%, (d) 40% and (e) 100% B1 weight ratios. Corresponding AFM phase images (f-j).

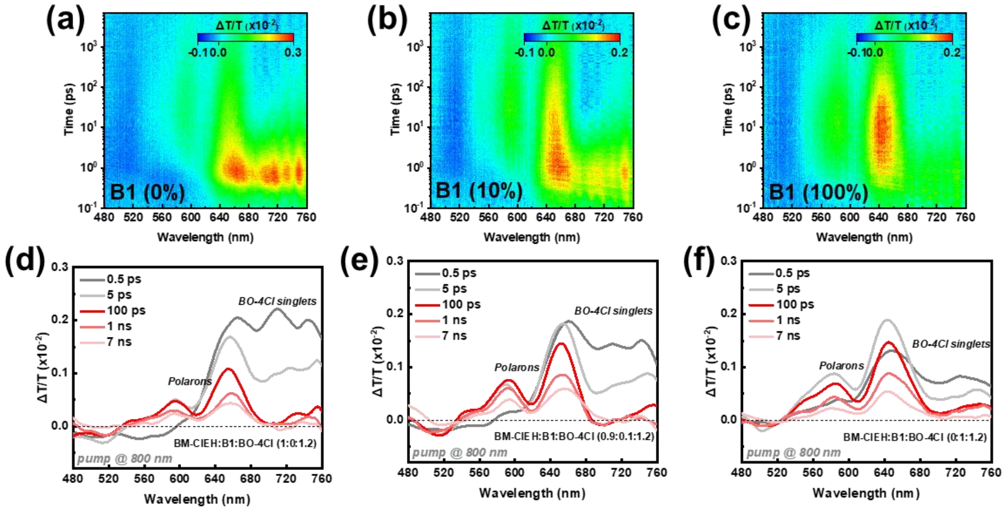

Supplementary Figure 33 2D plots of TAS measurements for BM-ClEH:B1:BO-4Cl (THF) blend films with (a) 0%, (b) 10% and (c) 100% B1 weight ratio. Spectral cuts of TAS measurements after different time for BM-ClEH:B1:BO-4Cl (THF) blend films (d) 0%, (e) 10% and (f) 100% B1 weight ratio.

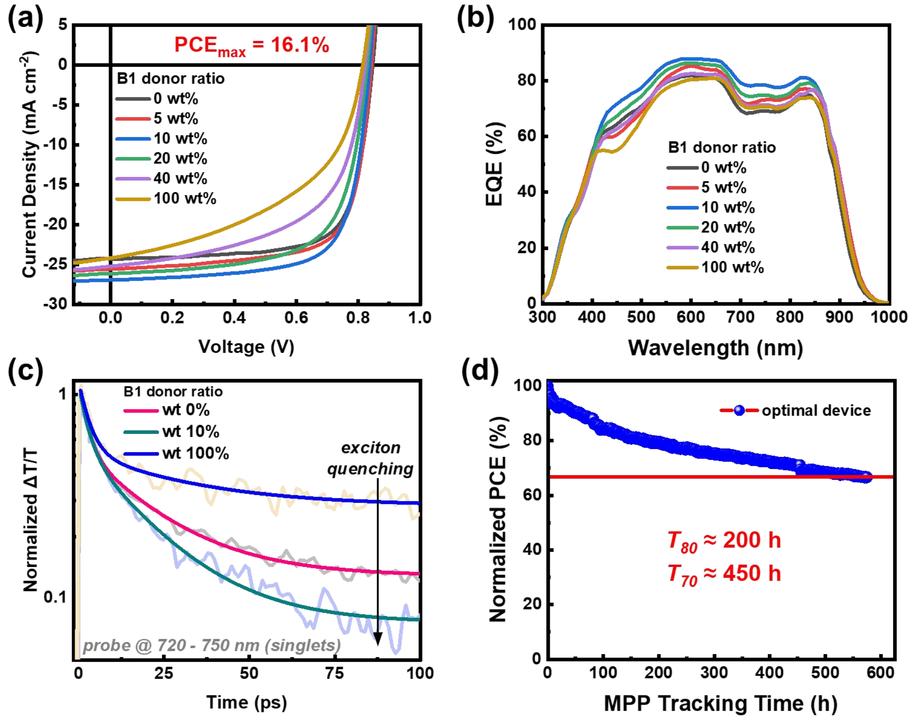

Supplementary Figure 34 (a) *J-V* curves of THF-processed ternary AMS-OSCs based on BM-ClEH:B1:BO-4Cl with different B1 weight ratio. (b) Corresponding EQE spectra. (c) Free charge generation and recombination kinetics of BM-ClEH:B1:BO-4Cl devices with 0%, 10% and 100% B1 addition. (d) Stability of

optimal device under MPP tracking.

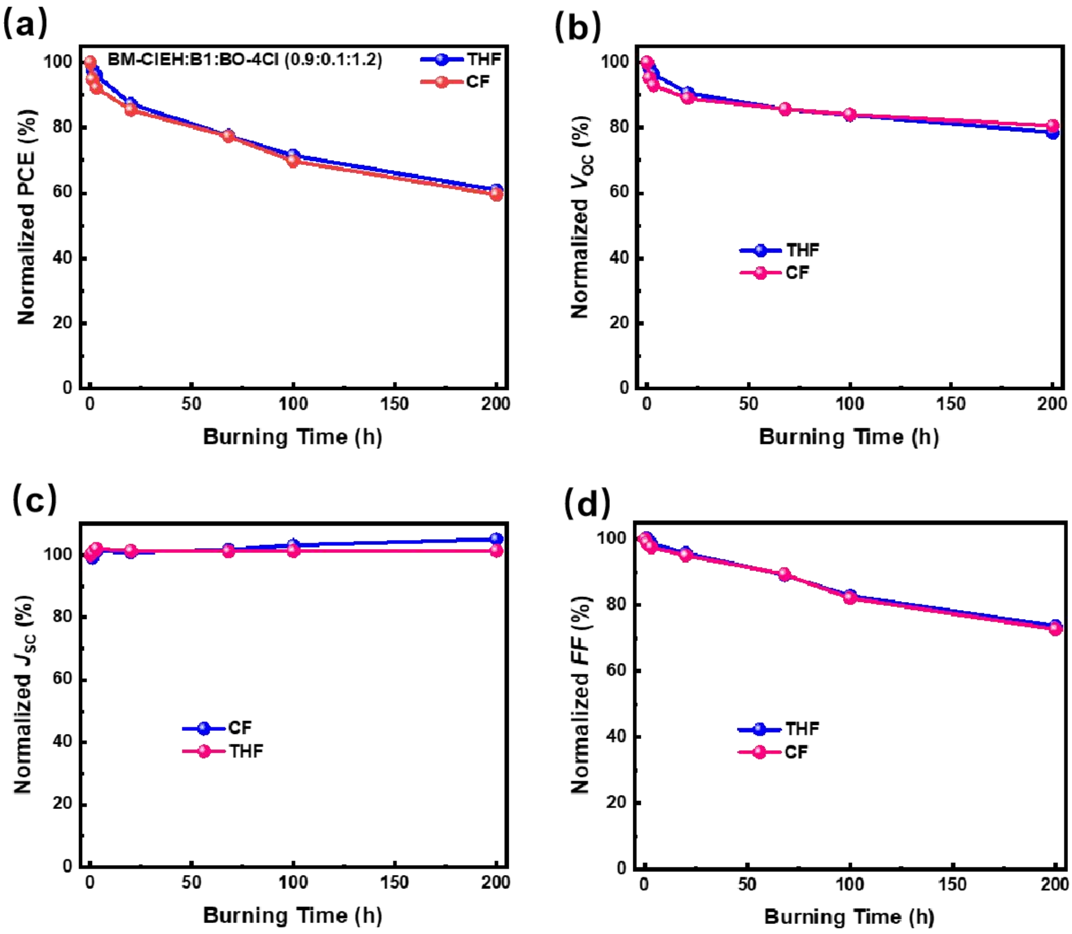

Supplementary Figure 35 Thermal stability of CF and THF-processed ternary devices based on BM-ClEH:B1:BO-4Cl (0.9:0.1:1) under a continuous TA at 80°C in a N_2 glovebox: (a) PCE; (b) V_{OC}; (c) J_{SC} and (d) FF.

NMR and MALDI-TOF-MS spectra

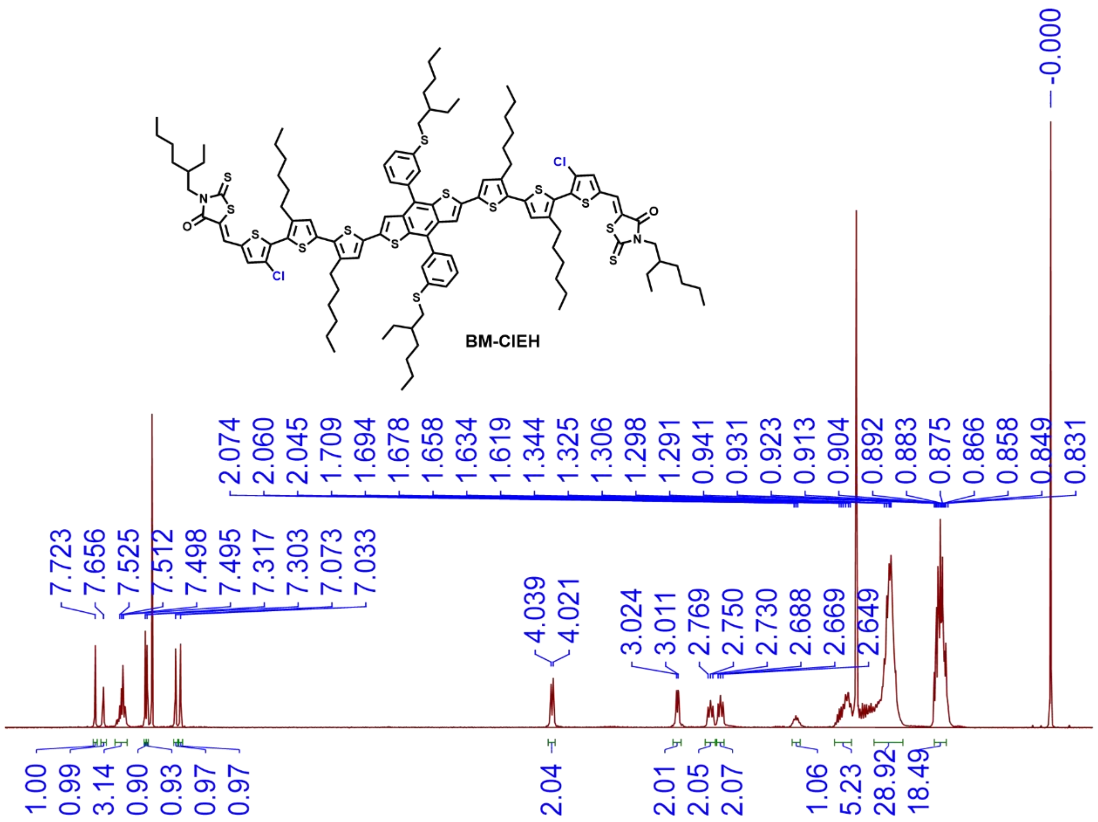

Supplementary Figure 36 ^{1}H NMR spectrum of BM-ClEH.

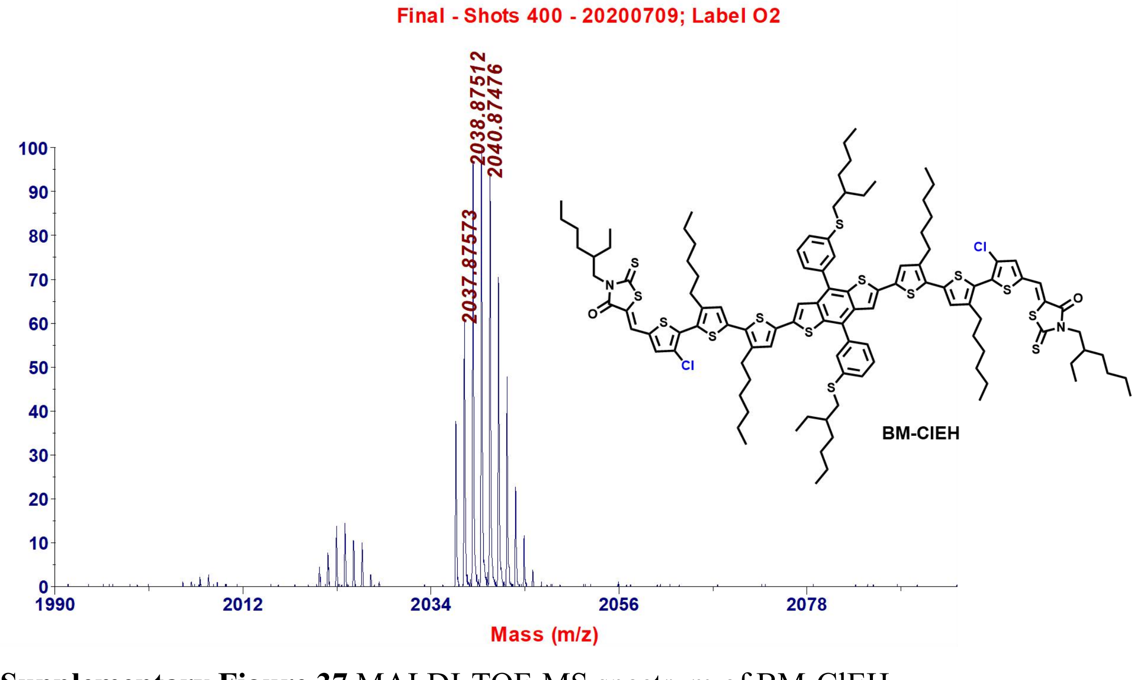

Supplementary Figure 37 MALDI-TOF-MS spectrum of BM-ClEH.

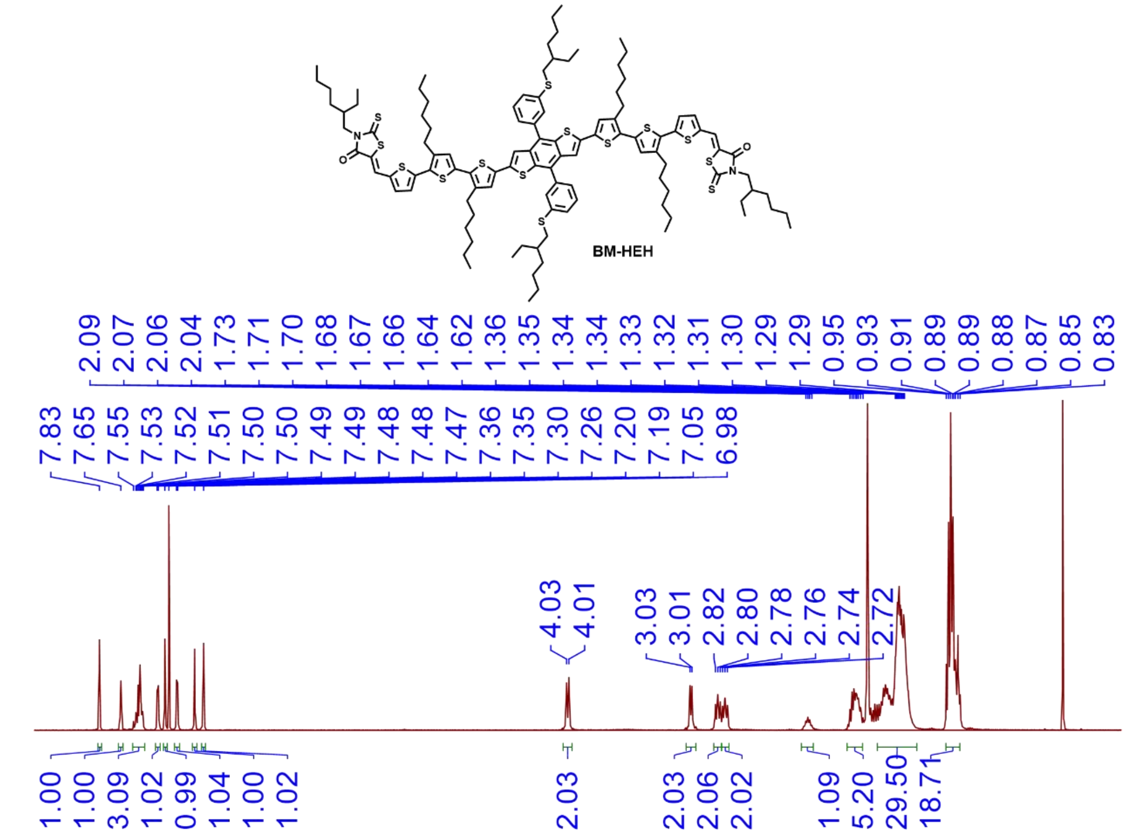

Supplementary Figure 38 ^{1}H NMR spectrum of BM-HEH.

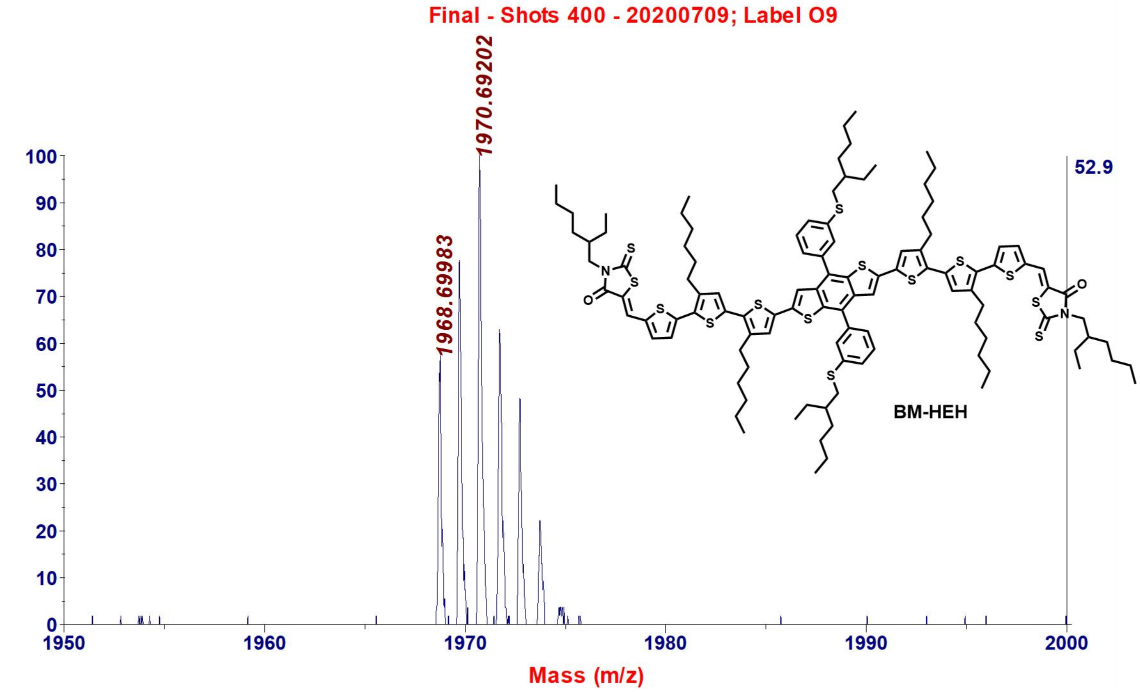

Supplementary Figure 39 MALDI-TOF-MS spectrum of BM-HEH.

Supplementary References

[1] Gao, W. et al. Intramolecular Chloro-Sulfur Interaction and Asymmetric Side-Chain Isomerization to Balance Crystallinity and Miscibility in All-Small-Molecule Solar Cells. *Angew. Chem. Int. Ed.* **61**, e202205168 (2022).

[2] Yang, L. et al. Modulating Molecular Orientation Enables Efficient Nonfullerene Small-Molecule Organic Solar Cells. *Chem. Mater.* **30**, 2129-2134 (2018).

[3] Qin, J. et al. 17% efficiency all-small-molecule organic solar cells enabled by nanoscale phase separation with a hierarchical branched structure. *Energy Environ. Sci.* **14**, 5903-5910 (2021).

[4] Heo, Y.-J. et al. Small-Molecule Organic Photovoltaic Modules Fabricated via Halogen-Free Solvent System with Roll-to-Roll Compatible Scalable Printing Method. *ACS Appl. Mater. Interfaces* **9**, 39519-39525 (2017).

[5] Burgués-Ceballos, I. et al. Solubility Based Identifi cation of Green Solvents for Small Molecule Organic Solar Cells. *Adv. Funct. Mater.* **24**, 1449-1457 (2014).

[6] Xiao, L. et al. Highly efficient small molecule solar cells fabricated with non-halogenated solvents. *RSC Adv.* **5**, 92312-92317 (2015).

[7] Cheng, X. et al. "Twisted" conjugated molecules as donor materials for efficient all-small-molecule organic solar cells processed with tetrahydrofuran. *J. Mater. Chem. A* **7**, 23008-23018 (2019).

[8] Liu, C. et al. Efficient Large Area All-Small-Molecule Organic Solar Cells Fabricated by Slot-Die Coating with Nonhalogen Solvent. *Adv. Funct. Mater.* 2300778(2023).
