# Supplementary material for: Efficient all-small-molecule organic solar cells processed with non-halogen solvent
Source: Nat Commun. 2024 Mar 2;15:1946. doi: 10.1038/s41467-024-46144-8 (PMC10908865; doi:10.1038/s41467-024-46144-8)
Supplement: Supplementary file 3 — Reporting Summary [file 41467_2024_46144_MOESM3_ESM.pdf]

nature research

Corresponding author(s): Gang Li

Solar Cells Reporting Summary

Nature Research wishes to improve the reproducibility of the work that we publish. This form is intended for publication with all accepted papers reporting the characterization of photovoltaic devices and provides structure for consistency and transparency in reporting. Some list items might not apply to an individual manuscript, but all fields must be completed for clarity.

For further information on Nature Research policies, including our data availability policy, see Authors & Referees.

Experimental design

Please check: are the following details reported in the manuscript?

1. Dimensions

Item	Yes/No	Explanation
Area of the tested solar cells	☒ Yes ☐ No	The device contact area was 0.042 cm2, device illuminated area during testing was 0.0324 cm2, which was determined by a mask. This information can be found in ASM-OSCs fabrication and characterization section.
Method used to determine the device area	☒ Yes ☐ No	The area of devices was determined by a mask. This information can be found in ASM-OSCs fabrication and characterization section.

2. Current-voltage characterization

Item	Yes/No	Explanation
Current density-voltage (J-V) plots in both forward and backward direction	☐ Yes ☒ No	The hysteresis effect of organic solar cells can be ignored, and the positive and negative scanning results are the same.
Voltage scan conditions<br>*For instance: scan direction, speed, dwell times*	☒ Yes ☐ No	The condition of voltage scanning is forward scanning at a speed of 0.01 V and dwell time of 1 ms.
Test environment<br>*For instance: characterization temperature, in air or in glove box*	☒ Yes ☐ No	The devices were characterized at room temperature in a glove box.
Protocol for preconditioning of the device before its characterization	☐ Yes ☒ No	*Explain why this information is not reported/not relevant.*
Stability of the J-V characteristic<br>*Verified with time evolution of the maximum power point or with the photocurrent at maximum power point; see ref. 7 for details.*	☐ Yes ☒ No	*Explain why this information is not reported/not relevant.*

3. Hysteresis or any other unusual behaviour

Item	Yes/No	Explanation
Description of the unusual behaviour observed during the characterization	☐ Yes ☒ No	*Explain why this information is not reported/not relevant.*
Related experimental data	☐ Yes ☒ No	*Explain why this information is not reported/not relevant.*

4. Efficiency

Item	Yes/No	Explanation
External quantum efficiency (EQE) or incident photons to current efficiency (IPCE)	☒ Yes ☐ No	This information can be found in manuscript and Supplementary Information.
A comparison between the integrated response under the standard reference spectrum and the response measure under the simulator	☒ Yes ☐ No	This information can be found in ASM-OSCs fabrication and characterization section.
For tandem solar cells, the bias illumination and bias voltage used for each subcell	☐ Yes ☒ No	*Explain why this information is not reported/not relevant.*

5. Calibration

Item	Yes/No	Explanation
Light source and reference cell or sensor used for the characterization	☒ Yes ☐ No	This information can be found in ASM-OSCs fabrication and characterization section.
Confirmation that the reference cell was calibrated and certified	☒ Yes ☐ No	This information can be found in ASM-OSCs fabrication and characterization section.

Calculation of spectral mismatch between the reference cell and the devices under test

- [ ] Yes
- [x] No

Explain why this information is not reported/not relevant.

6. Mask/aperture

Size of the mask/aperture used during testing

- [x] Yes
- [ ] No

This information can be found in ASM-OSCs fabrication and characterization section.

Variation of the measured short-circuit current density with the mask/aperture area

- [ ] Yes
- [x] No

Explain why this information is not reported/not relevant.

7. Performance certification

Identity of the independent certification laboratory that confirmed the photovoltaic performance

- [ ] Yes
- [x] No

Explain why this information is not reported/not relevant.

A copy of any certificate(s)
Provide in Supplementary Information

- [ ] Yes
- [x] No

Explain why this information is not reported/not relevant.

8. Statistics

Number of solar cells tested

- [x] Yes
- [ ] No

20 devices. This information can be found in manuscript and Supplementary Information.

Statistical analysis of the device performance

- [x] Yes
- [ ] No

The average PCE were calculated from 20 devices for each OSC. This information can be found in manuscript and Supplementary Information.

9. Long-term stability analysis

Type of analysis, bias conditions and environmental conditions
For instance: illumination type, temperature, atmosphere humidity, encapsulation method, preconditioning temperature

- [x] Yes
- [ ] No

This information can be found in ASM-OSCs fabrication and characterization section.
